# Single-cell chromatin profiling reveals relapse-related priming in pediatric acute myeloid leukemia

**DOI:** 10.26508/lsa.202603746

**Published:** 2026-08-24

**Authors:** Boyu Cui, Minghui Lei, Liwei Liu, Mingxin Shu, Furong Yu, Kai Yang, Yin Pan, Yunhong Song

**Affiliations:** 1 Department of Medical Technology, Anhui Institute of Medicine, Hefei, China; 2 Department of Hematology, The First Affiliated Hospital, Zhejiang University College of Medicine , Hangzhou, Zhejiang, China; 3 Key Laboratory of Hematopoietic Malignancies, Diagnosis and Treatment, Zhejiang, China; 4 School of Biological and Environmental Engineering, Chaohu University , Hefei, China

## Abstract

Relapse remains the leading cause of treatment failure and mortality in pediatric acute myeloid leukemia (pAML), yet the early molecular features underlying relapse susceptibility remain poorly understood. Here, we leveraged our previously generated single-cell chromatin accessibility dataset comprising 177,500 cells from 16 diagnostic pAML samples with long-term clinical outcome data, spanning t(8;21), inv(16), and FLT3-ITD subtypes and stratified into relapse (RPS) and non-relapsed (NRPS) cases. We show that RPS patients harbor relapse-associated chromatin accessibility signatures already detectable at diagnosis across multiple molecular subtypes. These patients display heightened innate immune and inflammatory activation and expansion of HSC/MPP-like leukemic progenitors with stem cell-like regulatory features. Motif enrichment analyses identify AP-1 family members, together with RUNX1, SPI1, and ETS factors, as central regulators shaping the relapse-associated epigenetic state. Elevated expression of these regulators predicts inferior survival across independent AML cohorts. Collectively, these findings suggest that early epigenetic priming of innate immune and inflammatory programs is associated with a relapse-prone state and highlight this core transcriptional network as a candidate biomarker framework in pAML.

## Abstract (precis)

This study identifies diagnosis-stage chromatin accessibility features associated with relapse-prone pediatric AML across molecular subtypes.

## Introduction

Pediatric acute myeloid leukemia (pAML) is a heterogeneous hematologic malignancy for which survival has improved modestly in recent years. Despite intensive chemotherapy, relapse remains the leading cause of treatment failure and mortality, with 30–40% of newly diagnosed patients ultimately experiencing disease recurrence (Ramos et al, 2018; Egan & Tasian, 2023; Lee et al, 2023). Unlike adult AML, which frequently accumulates age-associated mutations in genes such as *DNMT3A*, *TET2*, and RNA splicing factors (Bischof et al, 2024), pAML exhibits a comparatively restricted mutational landscape. Genomic alterations are more commonly characterized by recurrent core-binding factor rearrangements, such as RUNX1::RUNX1T1 and CBFβ–MYH11, and by kinase-activating lesions including *FLT3* internal tandem duplication (*FLT3*-ITD) (Karol et al, 2015; Hoffman et al, 2021; Yamato et al, 2022). Although these lesions provide diagnostic and prognostic value, they inadequately explain relapse risk in a substantial subset of patients, suggesting that additional non-genetic mechanisms may contribute to disease evolution and therapeutic failure (Abou Najem et al, 2019; Lambo et al, 2023).

Accumulating evidence indicates that epigenetic alterations play central roles in leukemogenesis, leukemia stem cell (LSC) maintenance, chemoresistance, and relapse (Xu et al, 2019; Agrawal-Singh et al, 2023). Key epigenetic regulators—including the mutated DNA methyltransferase DNMT3A, the m^6^A RNA demethylase ALKBH5, and the histone methyltransferase EZH2—have been implicated in sustaining stemness programs and promoting leukemic progression (Wong et al, 2019; Wang et al, 2020; Fang et al, 2024; Yu et al, 2025). Aberrant activity of such factors can further remodel the bone marrow microenvironment by modulating inflammatory and immune regulatory pathways, thereby promoting a pro-inflammatory and immunosuppressive niche that favors leukemic survival and clonal expansion (Belizaire et al, 2023; Yao et al, 2024). In parallel, dysregulated transcriptional regulators such as the AP-1 family and RUNX1 disrupt normal myeloid differentiation programs and reinforce stemness-associated transcriptional networks (Rossetti & Sacchi, 2013; Assi et al, 2019b). Recent chromatin-profiling studies also demonstrate that relapse is accompanied by extensive epigenetic remodeling and convergent regulatory evolution across genetically distinct leukemic clones following chemotherapy (Nuno et al, 2024). However, a fundamental question remains unresolved: when and how relapse-associated epigenetic programs emerge during the course of pAML. Specifically, it is unclear whether these states arise as adaptive responses to treatment or are already embedded within leukemic cells at diagnosis, thereby predisposing certain patients to relapse.

Advances in high-throughput single-cell sequencing technologies now enable precise delineation of leukemic heterogeneity and regulatory states that are masked in bulk analyses (Pellegrino et al, 2018; Fan et al, 2023; Xie et al, 2024). In adult AML, single-cell lineage tracing has shown that relapse frequently originates from pre-existing hematopoietic stem and progenitor cell (HSPC)-like populations harboring early driver lesions, highlighting the importance of cellular hierarchy and epigenetic state in relapse evolution (Le et al, 2020; Nuno et al, 2024; Shahar Gabay et al, 2025). Using mitochondrial single-cell ATAC-seq (mtscATAC-seq), we previously constructed a comprehensive single-cell chromatin accessibility atlas of pediatric AML, revealing pervasive activation of innate immune signaling at diagnosis and marked down-regulation of MHC-II molecules at relapse within primitive leukemic populations (Cui et al, 2025). That previous study primarily defined the epigenetic and clonal architecture of pAML progression, emphasizing subtype heterogeneity, clonal evolution, and disease-stage chromatin remodeling. However, it did not systematically determine whether patients who subsequently relapse already exhibit distinct chromatin accessibility programs at diagnosis, a question central to understanding early epigenetic priming before clinical relapse.

Building on this framework, we leveraged our established single-cell chromatin accessibility atlas comprising 177,500 cells from 16 molecularly characterized diagnostic pAML samples, classified into relapse (RPS; n = 9) and non-relapsed (NRPS; n = 7) groups according to subsequent clinical outcome. Our analyses identify relapse-associated chromatin accessibility programs that are already detectable at diagnosis and observed across multiple molecular subtypes. This early epigenetic priming is characterized by enhanced activation of innate immune and inflammatory pathways, together with a selective expansion of HSC/MPP-like leukemic progenitors exhibiting stem cell–like regulatory features. Motif enrichment and transcription factor footprinting analyses further identified a core regulatory circuitry involving AP-1 family members cooperating with RUNX1, SPI1, ETS, and BACH transcription factors. Elevated activity of this network strongly associates with inferior clinical outcomes across independent cohorts. Collectively, our findings suggest that relapse-associated chromatin programs are detectable at diagnosis and may represent an early epigenetic state associated with subsequent disease recurrence.

## Results

### Single-cell chromatin accessibility profiling of RPS and NRPS pAML at diagnosis

**Figure 1.**
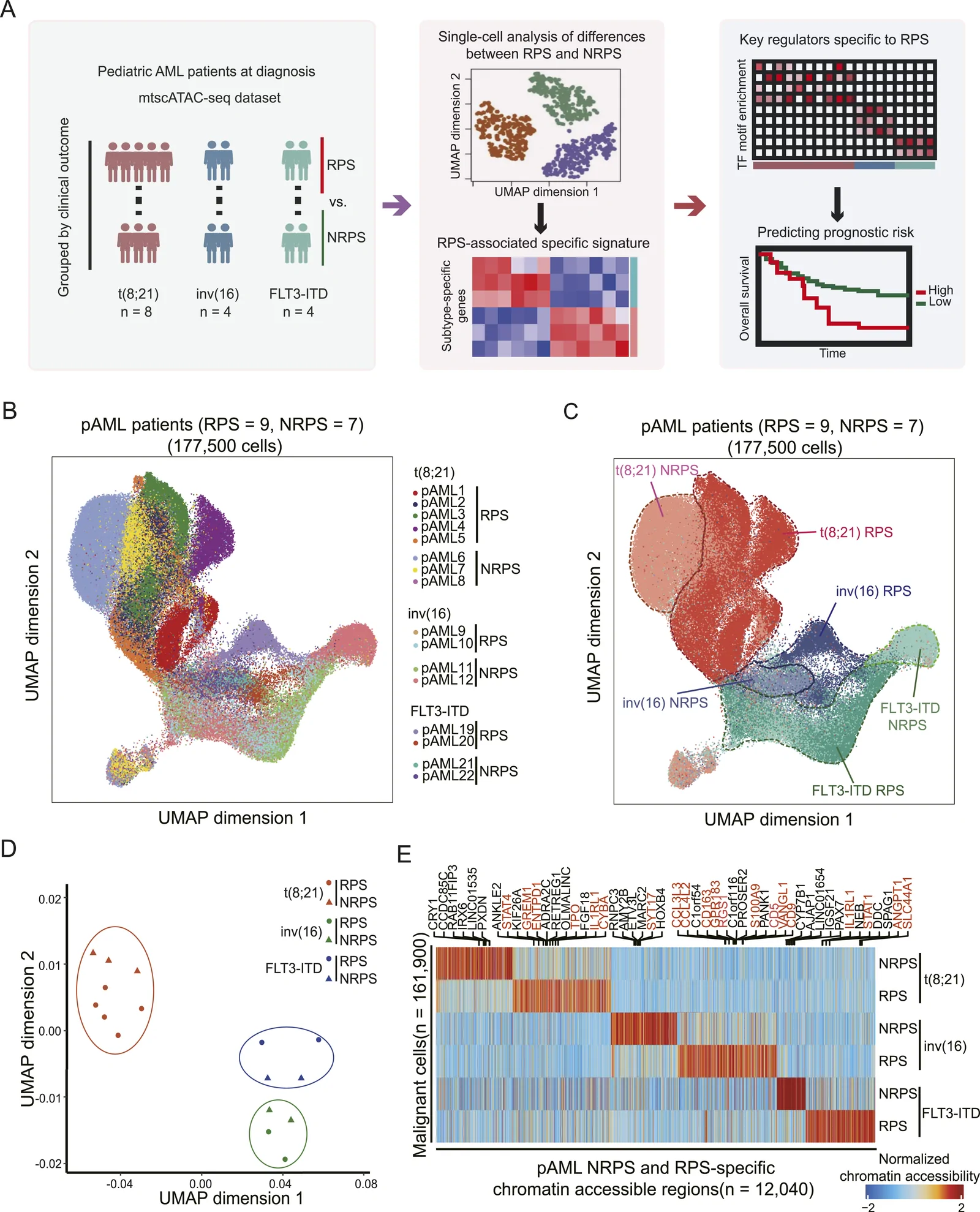
Single-cell chromatin accessibility profiling reveals relapse-associated epigenetic differences between RPS and NRPS pAML patients at diagnosis. **(A)** Schematic overview of the experimental workflow. Diagnostic bone marrow mononuclear cells and CD34^+^ leukemic cells from 16 pediatric AML (pAML) patients representing three molecular subtypes (t(8;21), inv(16), *FLT3*-ITD) were profiled to identify relapse-associated chromatin signatures. **(B)** UMAP visualization of 177,500 single cells from 16 pAML patients at single-cell level, with cells color-coded by individual samples. **(C)** UMAP visualization showing sample grouping by clinical outcome. Cells are colored by molecular subtype, with relapse (RPS) and non-relapsed (NRPS) cases indicated. **(D)** Pseudo-bulk clustering of each patient using the top 25,000 variable peaks. Colors denote pAML molecular subtypes. Squares denote RPS patients, whereas triangles represent NRPS patients. **(E)** Heatmap illustrating differentially accessible regions (n = 12,040) between RPS and NRPS patients within each molecular subtype. The color represents normalized chromatin accessibility. Representative genes associated with subtype and clinical outcome are annotated.

**Figure S1.**
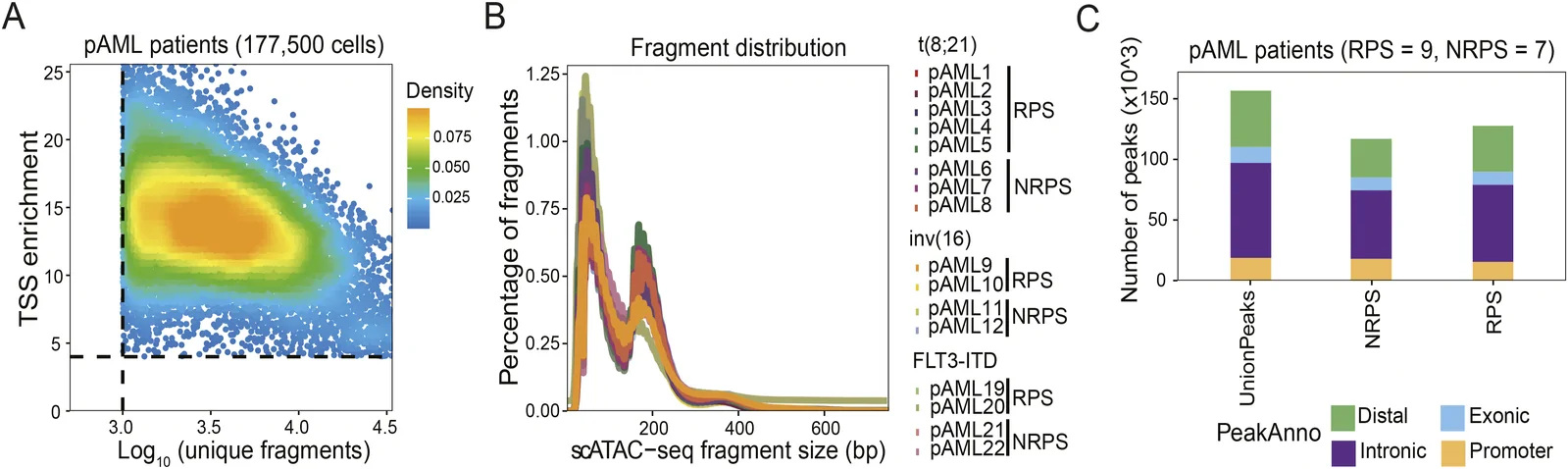
Quality control of pAML mtscATAC-seq dataset. **(A)** Transcription start site (TSS) enrichment score and unique nuclear fragments per cell. Dot color represents the density. **(B)** Fragment size distribution depicting characteristic periodicity attributed to nucleosome positioning across all samples. **(C)** Number and genomic annotation of unique peaks detected in RPS versus NRPS patients.

**Figure S2.**
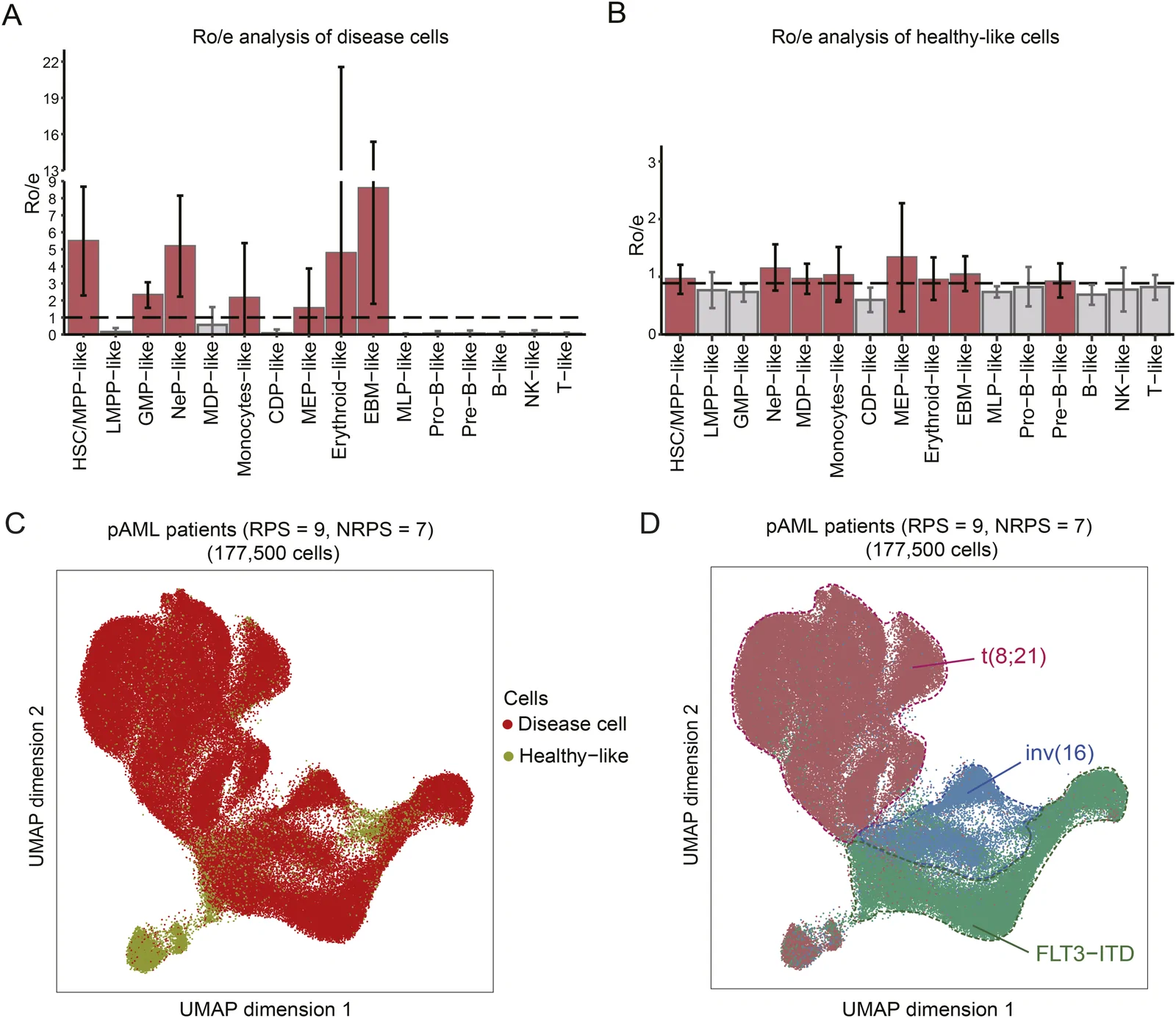
UMAP visualization and validation of malignant cell classification in the pAML dataset. **(A)** Ratio-of-observed-to-expected (Ro/e) analysis comparing the distribution of classified disease cells with healthy control hematopoietic cells across annotated cell types. Values close to 1 indicate comparable representation relative to healthy controls, whereas higher values indicate enrichment of the corresponding cell state in disease cells. **(B)** Ratio-of-observed-to-expected (Ro/e) analysis comparing the distribution of healthy-like cells from pAML samples with healthy control hematopoietic cells across annotated cell types. Values close to 1 indicate comparable representation relative to healthy controls. **(C)** UMAP embedding of single-cell chromatin accessibility profiles from pAML patients (n = 16). Cells are colored by accurately assigned disease/healthy-like states. **(D)** UMAP visualization of mtscATAC-seq pAML dataset, with cells color-coded by molecular subtype including t(8;21), inv(16) and *FLT3*-ITD at single-cell level.

To investigate whether epigenetic determinants of relapse are already established at diagnosis in pAML, we leveraged our previously established mtscATAC-seq pAML atlas (Cui et al, 2025) to perform an outcome-oriented comparison of chromatin accessibility states between relapse (RPS) and non-relapsed (NRPS) patients. The present study focused on 16 diagnostic pAML samples with available long-term clinical outcome information, together with the same eight healthy controls used as reference samples. The pAML cohort spanned three major molecular subtypes with distinct genetic and clinical features, including t(8;21) (RUNX1::RUNX1T1, n = 8), inv(16) (CBFβ–MYH11, n = 4), and *FLT3*-ITD (n = 4) (Fig 1A; Tables S1 and S2). In total, 177,500 high-quality single cells were profiled, encompassing both malignant and healthy-like hematopoietic populations. Quality control metrics, including transcription start site enrichment, fragment size distribution, and genomic annotation of detected peaks, are shown in Fig S1A–C. Malignant cells were identified using an integrated classification strategy established in our previously validated pAML mtscATAC-seq pipeline. This strategy incorporates leukemic genomic variants, copy-number signatures, chromatin accessibility patterns, and concordance with flow cytometric blast percentages. To further evaluate the reliability of this classification in the present cohort, we performed ratio-of-observed-to-expected (Ro/e) analyses comparing classified disease cells and healthy-like cells with healthy control hematopoietic cells. Healthy-like cells closely resembled healthy hematopoietic reference populations, whereas disease cells were preferentially enriched in primitive and progenitor-like compartments (Fig S2A and B). These results support the robustness of the malignant cell assignment used for downstream analyses. As expected, the cohort displayed substantial inter-patient heterogeneity, reflecting the intrinsic biological diversity of pAML. High-resolution single-cell analysis further revealed subtype-specific chromatin architectures across t(8;21), inv(16), and *FLT3*-ITD samples (Figs 1B and S2C and D). To minimize potential confounding from residual healthy-like hematopoietic cells, subsequent RPS versus NRPS chromatin accessibility analyses were restricted to confidently assigned malignant cells. Notably, direct comparisons of RPS and NRPS cases within the same molecular subtype uncovered clear differences in chromatin accessibility profiles at both aggregated (Fig 1C) and individual levels (Fig 1D). These observations suggest that RPS pAML samples harbor candidate relapse-associated epigenetic programs that are already detectable at diagnosis and are not solely attributable to molecular subtype.

Table S1. The information of pediatric healthy donors.

Table S2. Information on pediatric AML patients with available long-term clinical outcome data across molecular subtypes used in this study.

To define relapse-associated chromatin signatures across subtypes, we focused our analyses on malignant populations (161,900 cells). This revealed distinct and subtype-specific epigenetic patterns between RPS and NRPS patients. Accessible regions shared across both groups included loci associated with leukemogenesis and therapy resistance, such as *PXDN, CCDC85C, CYBA, RETREG1, FGF18, OLMALINC, HOXB4, RAB11FIP3, and CYP7B*, several of which (e.g., *HOXB4*, *CYBA*, *PXDN*) have been reported as prognostic biomarkers in AML (El-Meligui et al, 2022; Ijurko et al, 2022). In contrast, malignant cells from RPS patients exhibited a marked enrichment of accessible regions linked to innate immune and inflammatory signaling. These regions encompassed key regulatory genes including *TPO, STAT4, GREM1, ENTPD1, IL1RL1, S100A9, CCL3L3, CCL4L2, SLC44A1,* and *ANGPT1* (Fig 1E). Notably, *IL1RL1* and *CCL3L3* have been shown to promote inflammatory activation and support leukemia stem cell maintenance (Wang et al, 2019; Yao et al, 2021; Fu et al, 2025), whereas S100A9, frequently upregulated in MDS and AML, contributes to the establishment of a pro-inflammatory leukemic microenvironment (Cluzeau et al, 2017; Razmkhah et al, 2023). Collectively, these results indicate that RPS patients exhibit relapse-associated epigenetic alterations already present at diagnosis, extending beyond canonical oncogenic pathways to include regulatory elements governing immune and inflammatory dysregulation.

### Early divergence of cellular hierarchy and epigenetic activation in RPS patients

**Figure 2.**
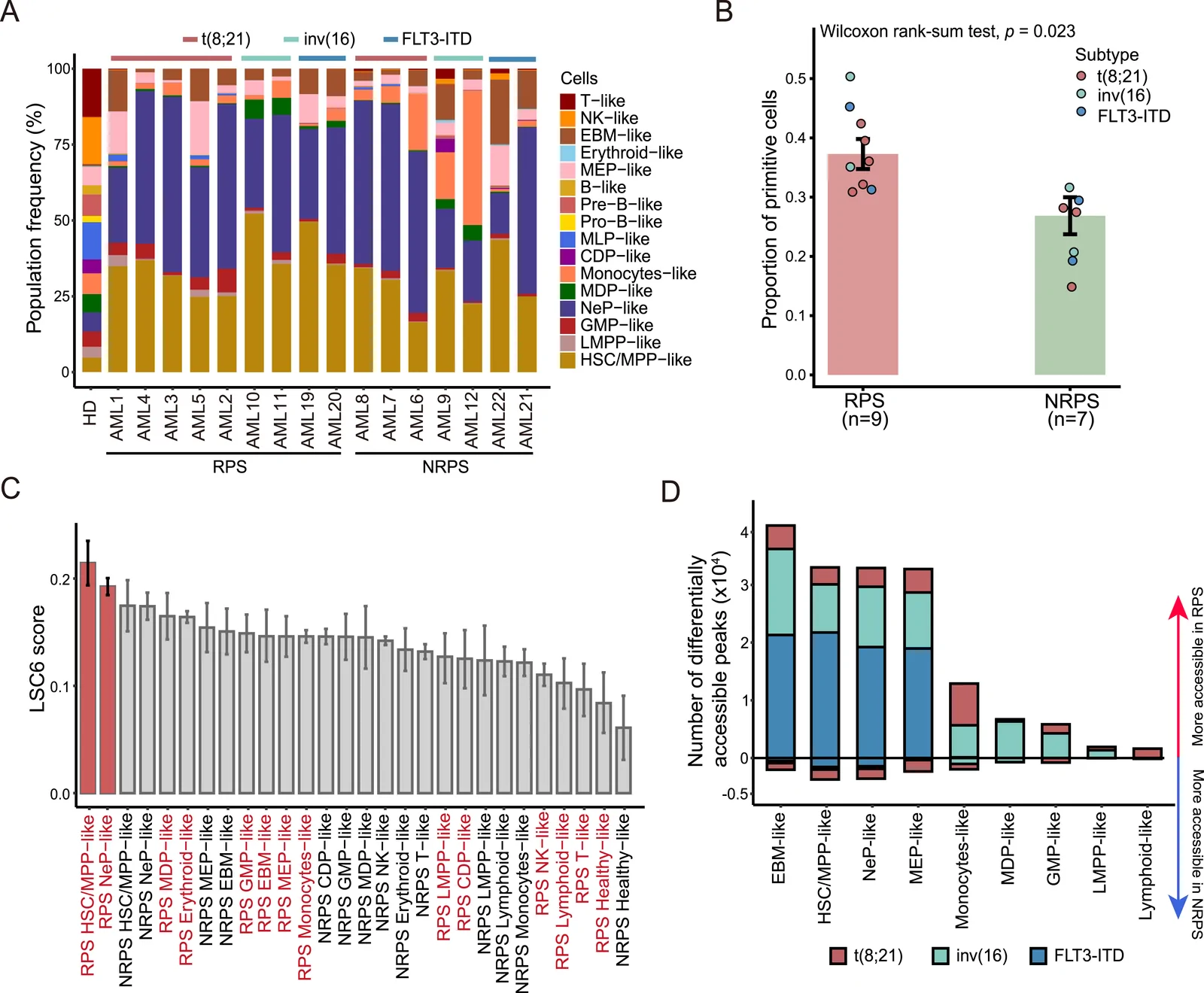
Early divergence of cellular hierarchy and chromatin accessibility patterns in RPS pAML. **(A)** Barplot showing the proportions of annotated malignant cell states across individual RPS and NRPS patients, with healthy donors (HDs) shown as a reference. RPS and NRPS samples are stratified by molecular subtype. **(B)** Comparison of the proportion of primitive blast populations between RPS and NRPS samples. Bars indicate mean values with standard error of the mean. Individual samples are overlaid and colored by molecular subtype. Statistical significance was assessed using the Welch’s *t* test. **(C)** Distribution of LSC6 gene scores across malignant cell types in RPS versus NRPS samples. **(D)** Barplot illustrating the number of differentially accessible peaks (DAPs) between RPS and NRPS samples across major progenitor-like subsets. Positive values indicate peaks more accessible in RPS samples, whereas negative values indicate peaks more accessible in NRPS samples. Colors indicate molecular subtypes.

**Figure S3.**
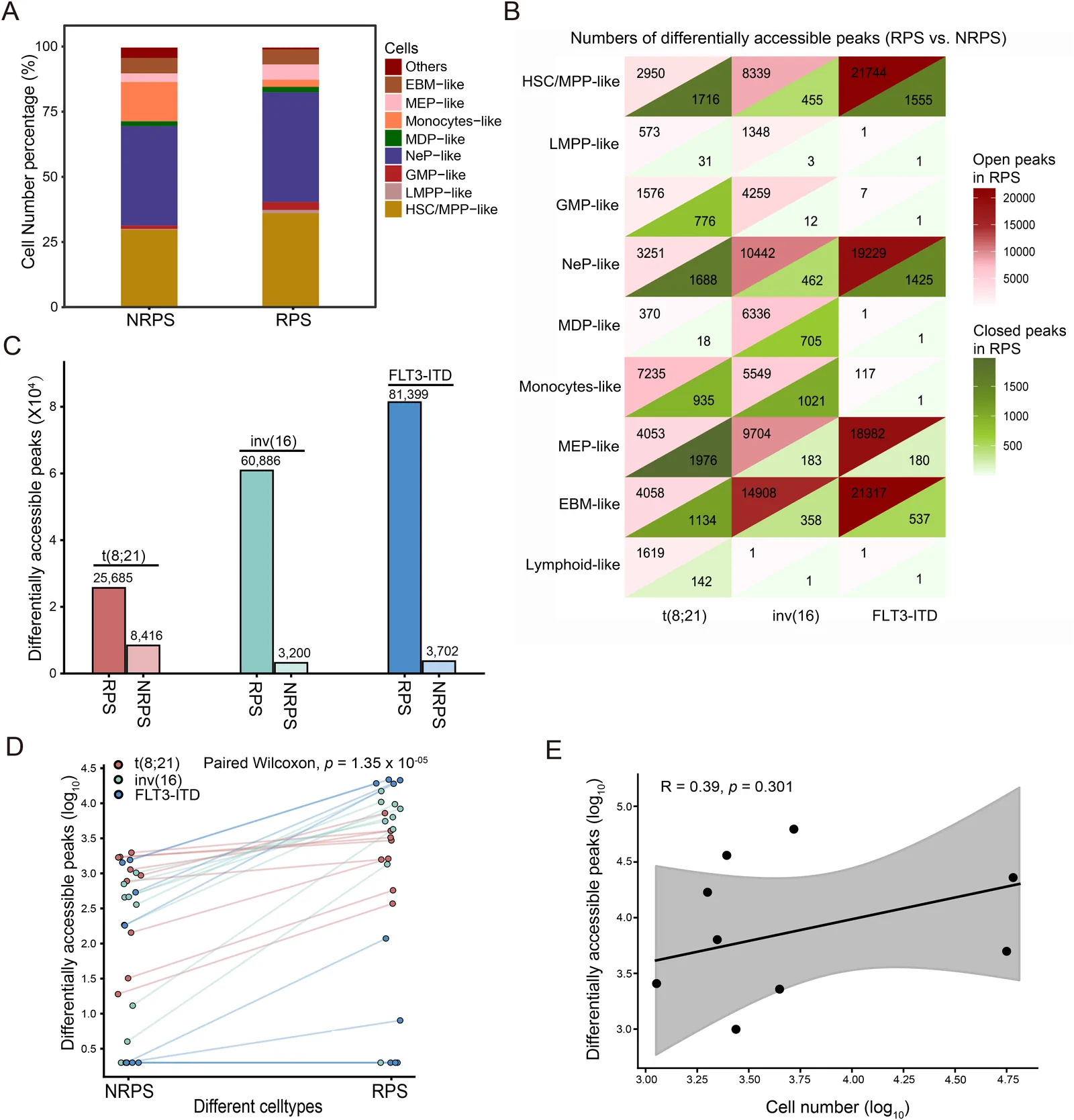
Comparison of malignant cell compositions and differentially accessible peak characteristics between RPS and NRPS samples. **(A)** Barplot showing the proportion of defined malignant cell types between RPS and NRPS groups. **(B)** Heatmap displaying the number of differentially accessible peaks per cell type between RPS and NRPS patients across molecular subtypes. The color scale represents the numbers of DAPs showing increased or decreased chromatin accessibility in RPS samples relative to NRPS patients. **(C)** Barplot showing the total numbers of DAPs more accessible in RPS or NRPS samples within each molecular subtype. For each subtype, the left bar indicates DAPs more accessible in RPS samples, and the right bar indicates DAPs more accessible in NRPS samples. **(D)** Paired comparison of DAP counts between peaks more accessible in RPS and peaks more accessible in NRPS across matched subtype–cell type pairs. Points and lines are colored by molecular subtype. Statistical significance was assessed using a paired Wilcoxon signed-rank test. **(E)** Scatter plot showing the correlation between the number of sequenced cells and number of differentially accessible peaks per sample (Spearman correlation with a 95% confidence interval for the regression line). The upper left corner displays the Spearman correlation coefficient (R) and the *P*-value.

We next sought to investigate relapse-associated early differences in leukemic cellular architecture. Using reference-based annotations from our previous pAML atlas (Cui et al, 2025), malignant cells were assigned to defined hematopoietic states. Both RPS and NRPS samples exhibited pronounced inter-patient heterogeneity and deviation from healthy hematopoiesis (Fig 2A), consistent with prior reports (Wiggers et al, 2019). However, clear distinctions in cellular architecture emerged between RPS and NRPS groups. RPS patients displayed a significantly higher proportion of primitive blast populations across three molecular subtypes, with an expansion of the HSC/MPP-like compartment (Figs 2B and S3A). Notably, both HSC/MPP-like and neutrophil progenitor-like (NeP-like) subsets in RPS samples exhibited higher LSC6 scores, a six-gene leukemia stem cell signature score previously developed to quantify LSC-like programs and identify high-risk pediatric AML patients (Elsayed et al, 2020). These findings indicate enrichment of stemness-associated regulatory features in RPS progenitor-like populations. In contrast, NRPS patients displayed reduced primitive burden and, in several cases, expansion of monocyte-like cells, reflecting partial preservation of myeloid differentiation (Fig 2A–C). These results indicate a fundamental divergence in disease architecture at diagnosis: RPS pAML preferentially engages LSC-like self-renewal programs with pronounced differentiation blockade.

We next compared chromatin accessibility patterns within matched malignant subsets. RPS samples showed a greater number of differentially accessible peaks (DAPs) than NRPS samples across multiple progenitor-like compartments, particularly among HSC/MPP-like, NeP-like, megakaryocyte-erythroid progenitor-like (MEP-like), and early basophil/mast progenitor-like (EBM-like) populations (Figs 2D and S3B). To assess whether these DAP patterns were primarily driven by a single molecular subtype, we stratified the analysis by molecular subtype and compared DAPs with higher accessibility in RPS or NRPS samples. DAPs with higher accessibility in RPS samples were more abundant than those with higher accessibility in NRPS samples across t(8;21), inv(16), and *FLT3*-ITD samples (Fig S3C). Consistently, paired analysis across matched subtype-cell type pairs showed higher DAP counts in RPS than in NRPS (Fig S3D), supporting the presence of relapse-associated chromatin accessibility differences across distinct molecular subtypes. Importantly, the number of DAPs showed non-significant association with the number of cells in the corresponding subsets (Fig S3E), suggesting that relapse-associated chromatin accessibility differences are unlikely to be explained solely by cell-type abundance and may partly reflect cell-intrinsic regulatory alterations. Thus, these results indicate that relapse-associated epigenetic remodeling is already detectable within specific progenitor-like populations at diagnosis and may underpin distinct pathological trajectories that determine clinical outcome.

### Early priming of innate immune and inflammatory signatures in RPS pAML

**Figure 3.**
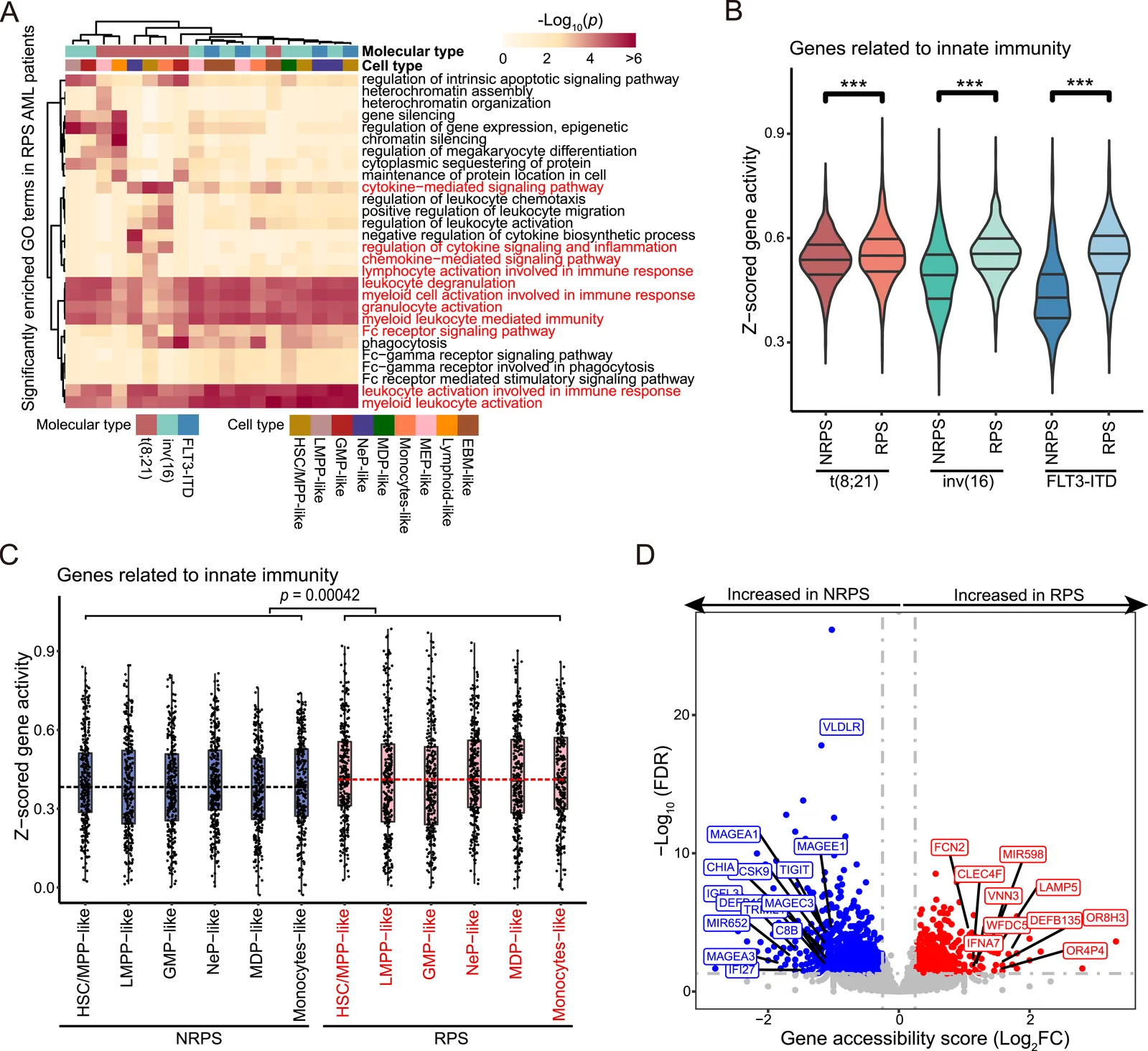
Heightened innate immune and inflammatory signaling in RPS pAML at diagnosis. **(A)** Heatmap of significantly enriched Gene Ontology (GO) terms derived from RPS-specific accessible regions across malignant cell types and subtypes. Color indicates enrichment significance. **(B)** Violin plot showing gene score of innate immune gene set (n = 334) in RPS and NRPS patients across distinct pAML subtypes. ***, *P* < 0.001, Wilcoxon rank-sum test. **(C)** Box plot showing gene score of innate immune gene set (n = 334) across major malignant cell populations in RPS versus NRPS patients. Statistical significance was assessed using the Wilcoxon rank-sum test. **(D)** Volcano plot showing differential gene accessibility between RPS (n = 66,481 cells) and NRPS (n = 55,222 cells) groups based on ArchR gene activity scores. Genes with FDR < 0.05 and |log_2_FC| > 0.1 were highlighted for visualization. Representative genes linked to innate immune and inflammatory signaling are labeled. Statistical significance was assessed using a Wilcoxon rank-sum test followed by Benjamini–Hochberg correction.

**Figure S4.**
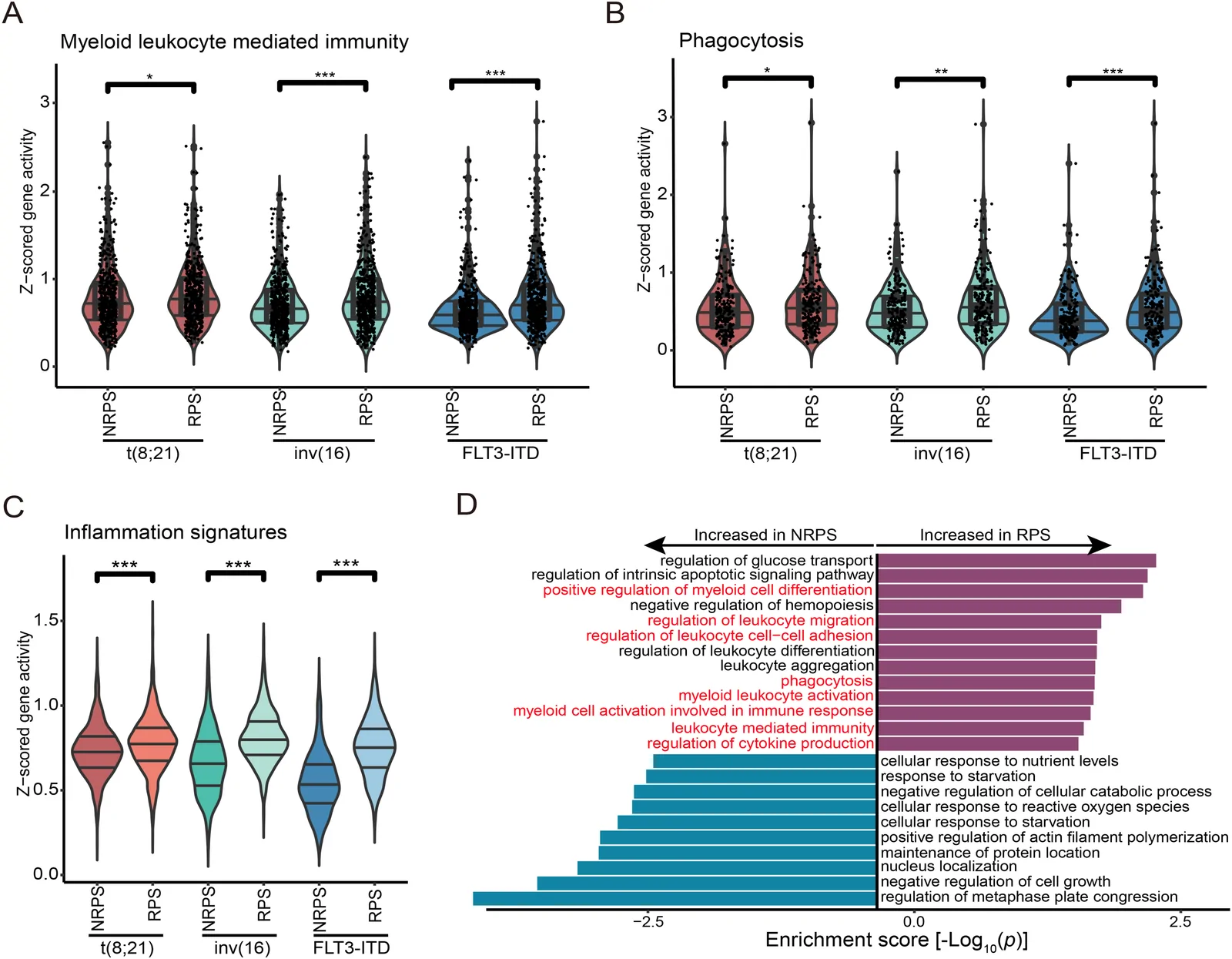
Comparison of inflammatory and immune-related features between RPS and NRPS pAML patients. **(A)** Violin plot showing Gene Ontology (GO) terms related to myeloid leukocyte mediated immunity in RPS and NRPS patients across distinct subtypes. *, *P* < 0.05; ***, *P* < 0.001; Wilcoxon rank-sum test. **(B)** Violin plot showing GO terms related to phagocytosis in RPS and NRPS patients across distinct subtypes. *, *P* < 0.05; **, *P *< 0.01, ***, *P* < 0.001; Wilcoxon rank-sum test. **(C)** Violin plot showing gene score of inflammation signatures gene set (n = 187) in RPS and NRPS patients across distinct pAML subtypes. ***, *P* < 0.001, Wilcoxon rank-sum test. **(D)** The significantly enriched GO terms derived from RPS- versus NRPS-specific accessible regions. Color indicates the significance of enrichment.

To evaluate the functional impact of relapse-associated chromatin alterations, we performed Gene Ontology (GO) enrichment analysis on DAPs. Compared with NRPS patients, RPS samples displayed marked enrichment for immune and inflammatory pathways, including cytokine/chemokine signaling, receptor-mediated activation, and myeloid cell migration (Fig 3A and B). These biological processes have been broadly implicated in leukemogenesis, inflammation-driven disease progression, and immune evasion (Hong et al, 2021; Luciano et al, 2022; Chen et al, 2024; Zhu et al, 2026). Importantly, this immune/inflammatory chromatin signature was consistently observed across multiple progenitor populations, including HSC/MPP-like, GMP-like, NeP-like, and MDP-like compartments (Fig 3A and C). Moreover, similar enrichment patterns were detected across different molecular subtypes of pAML (Figs 3A and B, and S4A–C), indicating that this inflammatory state is not restricted to a single genotype. These findings reveal that heightened innate immune and inflammatory activation represents a shared and defining epigenetic feature of RPS pAML.

Bulk-level aggregation further supported the single-cell results. RPS samples were preferentially enriched for pathways involved in hematopoiesis, myeloid differentiation, immune activation, and inflammatory signaling, whereas NRPS cases predominantly exhibited enrichment for metabolic adaptation, cellular stress responses, and mitotic regulation (Fig S4D). Notably, genes with increased accessibility in RPS samples were strongly linked to innate immune and inflammatory signaling (Fig 3D), such as *IFNA7*, *CLEC4F*, *FCN2*, *DEFB135,* and *WFDC5*, reinforcing the presence of an intrinsically activated immune state at diagnosis. Collectively, these results indicate that RPS samples exhibit an innate immune and inflammatory chromatin accessibility signature at diagnosis, suggesting that relapse-prone leukemic progenitors are epigenetically primed toward immune and inflammatory regulatory programs before therapy, which may be linked to relapse susceptibility in pAML.

### A relapse-associated transcription factor network predicts inferior survival

**Figure 4.**
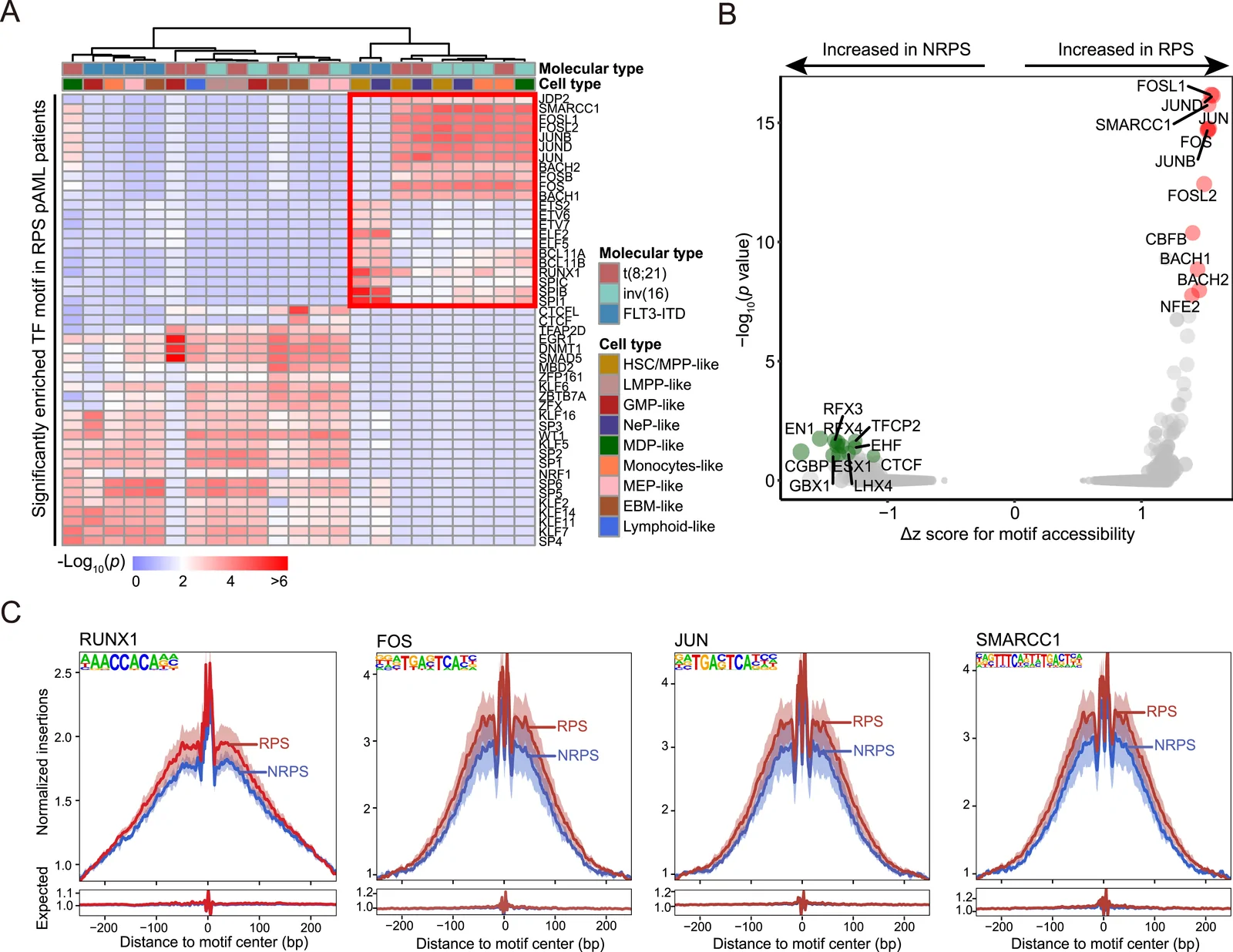
RPS leukemic progenitors display subtype-dependent activation of transcription factor (TF) regulatory programs. **(A)** Heatmap illustrating enriched TF motifs based on RPS-specific differentially accessible regions across selected progenitor-like populations. Top three motifs per cell type are shown, with color indicating enrichment significance. **(B)** Volcano plot of differential TF motif accessibility between RPS and NRPS samples. Positive Δz scores indicate increased motif accessibility in RPS, whereas negative Δz scores indicate increased motif accessibility in NRPS. Representative top motifs are labeled. Statistical analysis was performed using an LMM followed by a likelihood ratio test and Benjamini–Hochberg correction. **(C)** Aggregate ATAC-seq footprints for selected RPS-enriched TFs including RUNX1, FOS, JUN, and SMARCC1. Line colors indicate RPS (red) versus NRPS (blue) groups.

**Figure S5.**
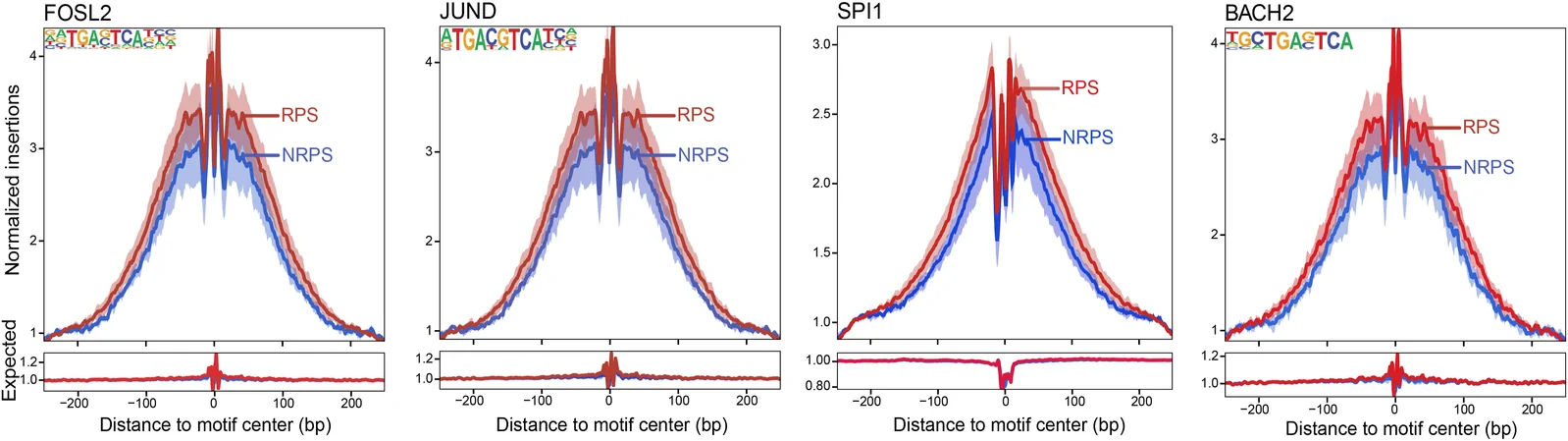
Aggregate TF footprint profiles for representative RPS-enriched TFs. Lines are colored by RPS and NRPS groups.

A. To identify upstream regulators driving the heightened innate immune and inflammatory epigenetic state in RPS patients, we performed motif enrichment analysis on RPS-specific DAPs. RPS samples exhibited strong enrichment of AP-1 family transcription factors (TFs), including JUN, FOS, FOSL1, FOSL2, and JUNB, particularly within HSC/MPP-like and NeP-like compartments of t(8;21) and inv(16) subtypes. In parallel, motifs associated with the BACH family—key regulators of hematopoietic differentiation and leukemogenesis—were significantly enriched, suggesting potential cooperative crosstalk with AP-1 in orchestrating inflammatory transcriptional programs. In *FLT3*-ITD RPS samples, motifs for ETS factors, RUNX1, BCL11A/B, and SPI1 were also preferentially enriched within HSC/MPP-like and NeP-like compartments (Fig 4A and B), indicating subtype-specific perturbations in lineage specification and hematopoietic differentiation. TF footprinting analysis further substantiated these observations by revealing increased local chromatin accessibility at binding sites for AP-1, BACH, RUNX1, and SPI1, consistent with elevated TF footprint signals in RPS leukemic cells (Figs 4C and S5). In addition to these lineage- and subtype-related regulators, multiple other TFs implicated in AML pathogenesis, such as CTCF, DNMT1, SP1, WT1, and members of the KLF family, also exhibited enrichment across additional RPS cell populations, reflecting their broad involvement in differentiation blockade and leukemic stem cell maintenance.

**Figure 5.**
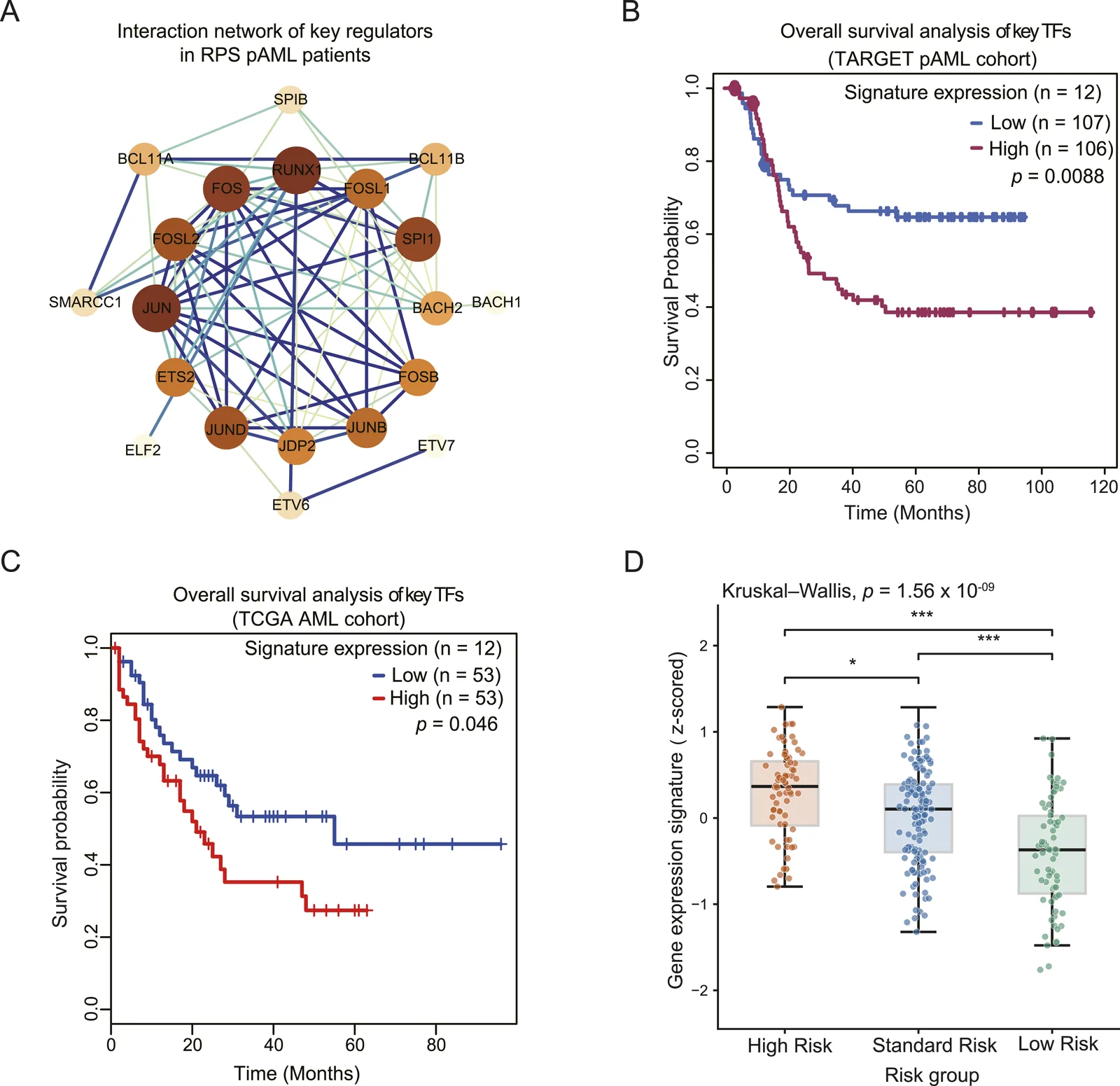
Core relapse-associated transcriptional regulators and their clinical relevance. **(A)** Protein–protein interaction (PPI) network constructed from RPS-enriched TFs. Node size reflects interaction degree; edge width and color represent interaction confidence. **(B)** Kaplan–Meier overall survival analysis in the TARGET pediatric AML cohort stratified by the key regulator signature. Patients were divided into high- and low-signature groups according to the median signature score (high versus low expression; high, n = 73; low, n = 72). Statistical significance was assessed using the log-rank test. **(C)** Kaplan–Meier overall survival analysis in the adult TCGA-LAML cohort stratified by the key regulator signature. Patients were divided into high- and low-signature groups according to the median signature score (high versus low expression; high, n = 53; low, n = 53). Statistical significance was assessed using the log-rank test. **(D)** Box plot showing the key TF expression signature score across TARGET pAML clinical risk groups. Statistical significance was assessed using the Kruskal–Wallis test, followed by Wilcoxon rank-sum tests.

**Figure S6.**
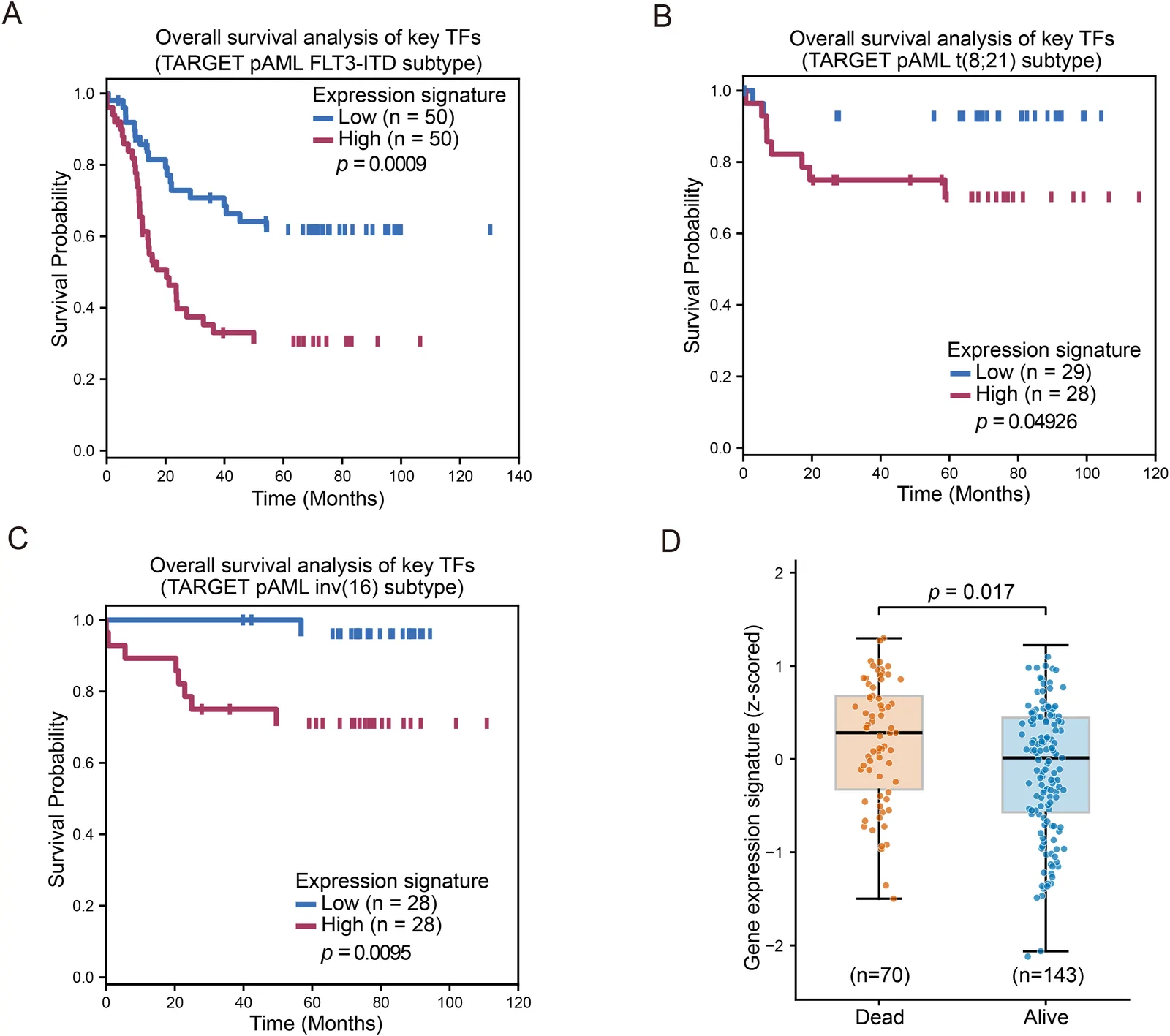
Subtype-specific survival analyses and clinical association of the relapse-associated TF signature in the TARGET pAML cohort. **(A, B, C)** Kaplan–Meier overall survival analyses of TARGET pAML patients with *FLT3*-ITD, t(8;21), and inv(16) subtypes stratified by the TF expression signature. Patients were divided into high- and low-signature groups according to the median signature score within each subtype. Statistical significance was assessed using the log-rank test. **(D)** Box plot showing the TF expression signature score in deceased and surviving patients from the TARGET pAML cohort. Statistical significance was assessed using the Wilcoxon rank-sum test.

To identify key regulators within this relapse-associated transcriptional circuitry, we constructed a protein–protein interaction (PPI) network from RPS-enriched TFs. AP-1 family members, RUNX1, SPI1, and JDP2, an AP-1-associated bZIP transcriptional regulator, emerged as highly interconnected hub nodes (Fig 5A), suggesting these factors function as core coordinators of the regulatory network in RPS samples. These TFs have been implicated in AML-related differentiation blockade, inflammatory signaling, and stress responses (Assi et al, 2019b; Granja et al, 2019; Fischer et al, 2024). We next evaluated the clinical association of this TF network using RNA-seq data from independent AML cohorts. A TF expression signature score was calculated based on the key regulators identified from motif enrichment, footprinting, and PPI network analyses. Kaplan–Meier survival analyses showed that high expression of this TF signature was associated with inferior overall survival in both the TARGET pediatric AML cohort (Fig 5B) and an independent TCGA AML dataset (Fig 5C), underscoring the robust clinical relevance of this regulatory network. Subtype-stratified Kaplan–Meier analyses in the TARGET cohort further showed that higher TF signature scores were associated with poorer survival within *FLT3*-ITD, inv(16), and t(8;21) subgroups (Fig S6A–C), suggesting that this survival association was not solely driven by a single molecular subtype. In addition, the TF expression signature score was significantly elevated in high-risk patients compared with standard-risk and low-risk patients (Fig 5D), and deceased patients showed higher TF expression signature scores than surviving patients in the TARGET pAML cohort (Fig S6D). Collectively, these analyses identify a relapse-associated TF network supported by chromatin accessibility, motif enrichment, footprinting, PPI network, and independent transcriptomic cohort analyses, and nominate this network as a candidate regulatory feature associated with adverse clinical outcomes in AML.

## Discussion

In this study, we identify relapse-associated epigenetic features that are already detectable at diagnosis in pAML patients who subsequently experience relapse. High-resolution single-cell chromatin accessibility profiling across major molecular subtypes reveals that RPS cases are characterized by heightened innate immune and inflammatory activation, accompanied by an expansion of HSC/MPP-like leukemic progenitors enriched for stemness-associated regulatory programs. These findings suggest that relapse susceptibility in pAML is associated with pre-existing epigenetic differences at diagnosis, rather than being explained solely by therapy-induced selection. The presence of similar inflammatory–stemness features across genetically distinct RPS cases further supports a relapse-associated regulatory axis that extends beyond individual molecular subtypes. Increased accessibility at immune-responsive chromatin regions may render RPS progenitors more responsive to inflammatory microenvironmental cues, potentially contributing to their persistence under therapeutic pressure.

At the regulatory level, we identify a core transcriptional network involving AP-1 family members (including JUN, FOS, FOSL1/2), RUNX1, SPI1, ETS factors, BACH2, and JDP2 as central regulators of this relapse-associated epigenetic architecture. AP-1 has been widely implicated in inflammatory gene activation, cytokine signaling, and leukemic stress adaptation (Trop-Steinberg & Azar, 2017), whereas RUNX1 and SPI1 are essential hematopoietic regulators whose dysregulation contributes to impaired differentiation and stem-like cell maintenance (Zhang et al, 2015; Assi et al, 2019b; Takei & Kobayashi, 2019). The cooperative motif enrichment and increased TF occupancy in RPS progenitors support a model in which AP-1 integrates inflammatory cues and synergizes with lineage-determining TFs to activate aberrant transcriptional programs that promote leukemic propagation and therapeutic resistance. Importantly, the identification of BACH family members and JDP2 within this regulatory module suggests that AP-1-centered programs in RPS pAML may involve broader transcriptional cooperation with additional hematopoietic and stress-responsive regulators. In addition, the association of this TF network with adverse outcomes in both TARGET pAML and TCGA-LAML cohorts suggests potential clinical relevance beyond pediatric AML.

Subtype-specific features refine these mechanistic insights. In core-binding factor (CBF) leukemias t(8;21) and inv(16), AP-1-driven regulatory programs dominate within immature progenitors, consistent with prior evidence that CBF fusions reshape enhancer architecture and sensitize cells to inflammatory stimuli (Al-Harbi et al, 2020; Bao et al, 2026). By contrast, *FLT3*-ITD RPS cases preferentially enrich for ETS, BCL11A/B, RUNX1, and SPI1 motifs, reflecting the intersection between hyperactive *FLT3* kinase signaling and lineage-specifying transcriptional networks through MAPK and STAT pathways (Assi et al, 2019a). Notably, the relapse-associated TF signature showed a particularly strong association with inferior survival in the *FLT3*-ITD subgroup, consistent with the marked enrichment of RPS-associated DAPs in this subtype. These findings suggest that *FLT3*-ITD leukemic cells may be especially susceptible to relapse-associated inflammatory and lineage-specifying chromatin states. Together, these observations provide an epigenetic rationale for the adverse clinical behavior of *FLT3*-ITD pAML and support the idea that inflammatory reprogramming may represent an underappreciated contributor to relapse susceptibility in this subtype.

Our results have several important translational implications. First, the robust and consistent presence of immune/inflammatory chromatin activation in RPS cases suggests that such signatures may represent candidate biomarkers for improving risk stratification at diagnosis, particularly for patients lacking high-risk genetic lesions. Because many of these TF programs can be reliably quantified in bulk samples, the AP-1/ETS/RUNX1/SPI1/JDP2 regulatory module could be explored in clinically scalable assays. Second, these findings suggest new therapeutic avenues. Inhibitors targeting AP-1-associated upstream pathways, including JNK/MAPK, TLR signaling, or IL-1/TNF axis, may diminish inflammatory priming and sensitize LSC-like populations to chemotherapy. Combining *FLT3* inhibitors with agents targeting inflammatory signaling represents a rational strategy for *FLT3*-ITD AML, which often fails to achieve sustained remissions due to adaptive epigenetic resistance (Scholl et al, 2020; Capelli et al, 2022). Finally, relapse-associated accessibility signatures derived from single-cell data may inform early therapeutic escalation, potentially enabling preemptive intervention in high-risk patients. Several limitations should be acknowledged. Although the cohort encompasses major pediatric AML subtypes, the sample size remains modest, and validation in larger, multi-center cohorts will be required to assess generalizability. In addition, our analyses focus on diagnostic specimens, therefore, the temporal evolution of these epigenetic programs during treatment and relapse remains unknown. Longitudinal multi-omics profiling, ideally incorporating clonal tracking, protein-level validation, and functional perturbation, will be essential to dissect dynamic remodeling, clarify the causal role of these regulators, and distinguish persistent relapse-fated clones from transient inflammatory states.

In summary, our study identifies a diagnosis-stage, relapse-associated epigenetic state in pAML characterized by innate immune and inflammatory activation and coordinated by a core transcriptional network spanning AP-1, RUNX1, SPI1, ETS, BACH, and JDP2. These findings provide regulatory insight into relapse susceptibility, highlight the importance of inflammatory epigenetic priming in shaping leukemic stem-like states, and nominate immune/inflammatory pathways and the associated TF regulatory network as candidate biomarkers and potential therapeutic vulnerabilities in pAML.

## Materials and Methods

### Patient samples and datasets

The single-cell chromatin accessibility data analyzed in this study were generated in our previously published pAML mtscATAC-seq atlas. Human sample collection and data use were approved by the Ethics Committee of Blood Diseases Hospital, Chinese Academy of Medical Sciences, as described in our previous study (Cui et al, 2025). The datasets are publicly accessible through our pAML mtscATAC database (http://www.pzhulab.com/app/Pediatric_AML_mtscATAC) and the China National Genomics Data Center (accession number HRA006415). The present analysis focused on 16 diagnostic pAML samples with available long-term clinical outcome information, together with eight healthy controls used as reference samples. No new human specimens were collected for the present study. The pAML cohort included molecular subtypes relevant to clinical risk stratification, including t(8;21) (RUNX1::RUNX1T1, n = 8), inv(16) (CBFβ–MYH11, n = 4), and *FLT3*-ITD mutations (n = 4). Subtype classification was performed according to established cytogenetic and molecular criteria aligned with WHO guidelines. Patients were stratified according to subsequent clinical outcome into relapse samples (RPS; patients who experienced relapse within three years after remission) and non-relapsed samples (NRPS; patients who remained in sustained remission for at least five years). All samples were obtained at diagnosis before chemotherapy. Standard induction therapy was administered to all patients. Relapse was characterized by the re-emergence of ≥5% leukemic blasts in the bone marrow or the reappearance of blasts in peripheral blood after remission.

### Single-cell mitochondrial ATAC-seq library quality control and preprocessing

All sequencing libraries were generated on an Illumina NovaSeq 6000 platform with paired-end 50bp reads. Raw sequencing reads were demultiplexed using CellRanger-ATAC (version 2.0.0) with the “mkfastq” pipeline, followed by alignment to the human reference genome GRCh38. Quality control (QC) metrics were applied to filter out low-quality cells based on the following criteria: (i) fewer than 1,000 or more than 50,000 unique nuclear fragments; (ii) transcription start site (TSS) enrichment score less than 3; (iii) fragment counts contributing more than 5% within genomic blacklisted regions; (iv) nucleosome signal score more than 4; (v) doublet score greater than 0.5, as determined by ArchR’s addDoubletScores function. Cells that met all these quality thresholds were retained for downstream analysis. After filtering, high-quality cells were retained and normalized for fragment depth. Potential doublets were identified via the “addDoubletScores” function and subsequently removed using the “filterDoublets” function within the ArchR framework (version 1.0.1). Following QC filtering, a total of 177,500 high-quality single cells were obtained across all samples. The aligned data were then processed using the ArchR pipeline for subsequent analyses.

### Malignant cell classification

Malignant cell classification was performed using the reference-based strategy established in our previous pAML mtscATAC-seq atlas (Cui et al, 2025). Briefly, single-cell chromatin accessibility profiles from pAML samples were projected onto a pediatric healthy hematopoietic reference map to distinguish disease cells from healthy-like hematopoietic cells. Cells that deviated from normal hematopoietic chromatin states and clustered within leukemia-associated regions were classified as disease cells, whereas cells mapping to normal hematopoietic compartments were classified as healthy-like cells. To further assess the classification, ratio-of-observed-to-expected (Ro/e) analyses were performed by comparing the distribution of classified disease cells or healthy-like cells with healthy control hematopoietic cells across annotated cell types. Additional validation of malignant cell assignment, including leukemia-associated genomic signals, copy-number alterations, AML-related chromatin signatures, and concordance with clinical blast percentages, was performed in our previous pAML atlas.

### Dimensionality reduction, clustering, and peaks calling

All single-cell chromatin accessibility analyses, including dimensionality reduction, clustering, and peak calling, were performed using the ArchR pipeline. Briefly, dimensionality reduction was conducted using Harmony to correct for batch effects across samples. Single-cell clustering was performed with the “addClusters” function in ArchR, and cluster identities were annotated using the “addGeneIntegrationMatrix” function by referencing a well-characterized, multimodal bone marrow mononuclear cell (BMMC) single-cell dataset (Cui et al, 2025). For visualization, latent semantic indexing (LSI) followed by uniform manifold approximation and projection (UMAP) was applied to represent chromatin accessibility profiles at the single-cell level. Peak calling was performed for each cell cluster or pAML subtype using the “addReproduciblePeakSet” function in ArchR with MACS2. Gene accessibility scores were computed based on chromatin accessibility within gene bodies, promoters, and distal regulatory regions. Malignant cell and cell type annotation were guided by our previously established pediatric healthy hematopoietic epigenetic reference map (http://www.pzhulab.com/app/Pediatric_AML_mtscATAC), representing the hematopoietic hierarchy at homeostasis, and further validated using canonical marker gene accessibility.

### Identification of differentially accessible peaks (DAPs)

Differential chromatin accessibility analysis between RPS and NRPS samples was conducted using the ArchR “getMarkerFeatures” function with a Wilcoxon rank-sum test, applying Benjamini–Hochberg false discovery rate (FDR) correction. DAPs were defined as peaks with |log_2_FC| ≥ 1.5 and FDR < 0.05 using the Wilcoxon rank-sum test.

### Gene ontology and pathway enrichment analyses

Genes associated with DAPs were mapped to nearest transcription start sites (TSS ± 2 kb). Functional enrichment analyses were performed using ClusterProfiler (v4.8.3) and the Reactome pathway database (http://www.reactome.org). Pathways with adjusted *P* < 0.05 were considered significantly enriched.

### Transcription factor motif and footprinting analyses

The “getMarkerFeatures” function in ArchR was used to identify differential peaks between RPS and NRPS groups. Peaks with significant differences, defined by FDR ≤ 0.05 and |log_2_FC| ≥ 1.5, were selected for motif enrichment analysis. To estimate motif enrichment in chromatin regions with differential accessibility, we used the chromVAR pipeline (v1.8.0). Hypergeometric testing followed by Benjamini–Hochberg correction was used to assess motif enrichment. Motifs with an adjusted *P*-value <0.01 were considered significantly enriched and were ranked according to enrichment z-score. To prioritize key TFs for downstream analyses, we focused on recurrently enriched motifs that were detected across multiple pAML patients. Footprinting analysis for the TFs of interest, with Tn5-bias correction, was performed by using the “plotFootprints” function within the ArchR pipeline to evaluate differential binding activity between RPS and NRPS samples, focusing on AP-1 (JUN, FOS, FOSL1/2, JUNB), RUNX1, SPI1, ETS, and BACH family members.

### Protein–protein interaction network construction

RPS-enriched transcription factors identified from motif analysis were used to construct a protein–protein interaction (PPI) network with the STRING (v12.0) database. Visualization and network topology analysis were conducted in Cytoscape (v3.9.1). Hub TFs were identified based on degree centrality and betweenness metrics. Only interactions with a STRING confidence score greater than 0.9—corresponding to high-confidence predictions (approximately 80–90% confidence)—were retained for network construction.

### Survival and TF expression signature analysis

To evaluate the prognostic and transcriptomic relevance of RPS-associated transcription factors, we analyzed gene expression and survival data from independent AML cohorts, including TARGET-AML as a pediatric cohort and TCGA-LAML as an adult cohort. Key TFs were selected based on motif enrichment, TF footprinting, and protein–protein interaction network analyses. For each patient, the TF expression signature score was calculated by z-score transformation of individual TF expression values followed by averaging across the selected TFs. Patients were divided into high- and low-signature groups according to the median signature score within each cohort or subtype. Kaplan–Meier survival curves were generated using the survival R package (v3.6.1), and statistical significance was assessed using the log-rank test. For clinical association analyses in TARGET pAML, TF expression signature scores were compared across clinical risk groups using the Kruskal–Wallis test. Differences in TF expression signature scores between deceased and surviving patients were assessed using the Wilcoxon rank-sum test.

### Statistical analysis

All statistical analyses were conducted in R (v3.6.1). Figures were generated using ggplot2, ComplexHeatmap, and EnhancedVolcano packages. Specific statistical tests, thresholds, and multiple testing corrections for each analysis are detailed in the Methods and figure legends for clarity.

## Supplementary Material

Reviewer comments

## Data Availability

Raw and processed mtscATAC-seq data analyzed in this study were generated and reported in our previous study (Cui et al, 2025) and are available through the China National Genomics Data Center under accession number HRA006415. The TARGET pediatric AML RNA-seq and clinical data analyzed in this study are available through the NCI Genomic Data Commons under the TARGET-AML project, with dbGaP accession phs000465. The TCGA-LAML RNA-seq and clinical data analyzed in this study are available through the NCI Genomic Data Commons under the TCGA-LAML project, with dbGaP accession phs000178. Additional processed data and code for reproducing analyses are available upon reasonable request to the corresponding author.
