# Supplementary material for: Single-cell chromatin profiling reveals relapse-related priming in pediatric acute myeloid leukemia
Source: Life Sci Alliance. 2026 Aug 24;9(11):e202603746. doi: 10.26508/lsa.202603746 (PMC13504080; doi:10.26508/lsa.202603746)
Supplement: Supplementary file 1 [file LSA-2026-03746_review_history.pdf]

Single-cell chromatin profiling reveals relapse-related priming in pediatric acute myeloid leukemia

Boyu Cui, Minghui Lei, Liwei Liu, Mingxin Shu, Furong Yu, Kai Yang, Yin Pan, and Yunhong Song
DOI: https://doi.org/10.26508/lsa.202603746

Corresponding author(s): Boyu Cui, Anhui Institute of Medicine and Yunhong Song, Chaohu University

**Review Timeline:**		
	Submission Date:	2026-04-22
	Editorial Decision:	2026-06-04
	Revision Received:	2026-07-03
	Editorial Decision:	2026-08-10
	Revision Received:	2026-08-11
	Accepted:	2026-08-11

Scientific Editor: Tim Fessenden

Transaction Report:

(Note: With the exception of the correction of typographical or spelling errors that could be a source of ambiguity, letters and reports are not edited. The original formatting of letters and referee reports may not be reflected in this compilation.)

June 4, 2026

Re: Life Science Alliance manuscript #LSA-2026-03746-T

Boyu Cui
Anhui Medical College

Dear Dr. Cui,

Thank you for submitting your manuscript entitled "Single-cell chromatin profiling reveals early relapse-associated epigenetic priming in pediatric acute myeloid leukemia" to Life Science Alliance. The manuscript was assessed by expert reviewers, whose comments are appended to this letter.

As you will see, reviewers were unanimous in their interest in this work and its potential significance for understanding relapse in pediatric leukemia. However, Reviewers 1 and 2 expressed important concerns with the main claims and key points of validation, which preclude publication of your work at this time. We invite you to submit a revised manuscript that addresses all points raised by Reviewers 1 and 2, some of which overlap. These include revising the major claims, including subtype-specific data in Figures 2 and 5, validating malignant vs normal cell assignment, and validating gene expression changes. Reviewer 3 made several suggestions to improve the text, which we invite you to consider.

I would be happy to discuss the revision in more detail via email or phone/videoconferencing. Please let me know which option you prefer, if any.

To upload the revised version of your manuscript, please log in to your account: https://lsa.msubmit.net/cgi-bin/main.plex

You will be guided to complete the submission of your revised manuscript and to fill in all necessary information. Please get in touch in case you do not know or remember your login name.

The typical timeframe for revisions is three months. Please note that papers are generally considered through only one revision cycle, so strong support from the referees on the revised version is needed for acceptance.

While you are revising your manuscript, please also attend to the below editorial points to help expedite the publication of your manuscript. Please direct any editorial questions to the journal office. When submitting the revision, please include a letter addressing the reviewers' comments point by point.

Thank you for this interesting contribution to Life Science Alliance. We hope that the comments below will prove constructive as your work progresses, and we are looking forward to receiving your revised manuscript.

Sincerely,

Tim Fessenden
Scientific Editor
Life Science Alliance

A. THESE ITEMS ARE REQUIRED FOR REVISIONS

-- A letter addressing the reviewers' comments point by point.

-- An editable version of the final text (.DOC or .DOCX) is needed for copyediting (no PDFs).

-- High-resolution figure, supplementary figure and video files uploaded as individual files: See our detailed guidelines for preparing your production-ready images, https://www.life-science-alliance.org/authors

-- Summary blurb (enter in submission system): A short text summarizing in a single sentence the study (max. 200 characters including spaces). This text is used in conjunction with the titles of papers, hence should be informative and complementary to the title and running title. It should describe the context and significance of the findings for a general readership; it should be written in the present tense and refer to the work in the third person. Author names should not be mentioned.

-- By submitting a revision, you attest that you are aware of our payment policies found here: https://www.life-science-alliance.org/copyright-license-fee

B. MANUSCRIPT ORGANIZATION AND FORMATTING:

Full guidelines are available on our Instructions for Authors page, https://www.life-science-alliance.org/authors

We encourage our authors to provide original source data, particularly uncropped/-processed electrophoretic blots and spreadsheets for the main figures of the manuscript. If you would like to add source data, we would welcome one PDF/Excel-file per figure for this information. These files will be linked as supplementary "Source Data" files.

IMPORTANT: It is Life Science Alliance policy that if requested, original data images must be made available. Failure to provide original images upon request will result in unavoidable delays in publication. Please ensure that you have access to all original microscopy and blot data images before submitting your revision.

Reviewer #1 (Comments to the Authors (Required)):

In this manuscript, the authors investigate early relapse-associated epigenetic features in pediatric acute myeloid leukemia (pAML). This is a very interesting and important question in pAML, especially due to its potential role in predicting relapse. This study uses data mining to investigate these claims and attempts to connect relapse to chromatin accessibility signatures at diagnosis, including enrichment of innate immune and inflammatory pathways, expansion of stem cell-like regulatory programs, and increased accessibility of motifs associated with AP-1, RUNX1, SPI1 and ETS transcription factors. However, some of the conclusions in this manuscript overlap with a previously published Blood paper (Cui et al. 2025), resulting in limited novelty. Without functional validation and larger datasets, some conclusions are overstated. The following points should be addressed prior to publication.
Major comments
(1) This manuscript appears to be a re-analysis of the authors' previously published pAML mtscATAC-seq dataset. While the re-analysis of existing data is valuable, the manuscript should clearly explain how it differs from the prior paper. Otherwise, the novelty is limited.
(2) This manuscript claims the relapse-associated signature is independent of molecular subtype. Considering the small sample size, it is not enough to strongly claim subtype independence.
(3) The correct malignant cell assignment is important for downstream analysis. The authors refer to their previous pAML atlas for reference-based annotation, but in this manuscript, it is still unclear how malignant cells were distinguished from normal cells, which may potentially contribute to the observed difference in chromatin accessibility.
(4) In the chromatin accessibility analysis beginning at line 208, the authors conclude that the observed differences reflect cell-intrinsic regulatory alterations rather than compositional effects. This conclusion is too strong. Although the reported correlation is not statistically significant, the limited sample size and moderate positive trend do not exclude the possibility that cell abundance contributes to the number of differentially accessible regions. The authors should explore other normalization methods.
(5) As written, the study identifies candidate relapse-associated molecular features rather than validated early predictors of relapse. The manuscript should therefore temper the use of "predictors" unless independent validation and multivariable predictive modeling are provided.
Minor comments
(1) Fig. 2D should clarify if the differentially peaks are more open or closed in RPS or NRPS.
(2) The relevance of the survival analyses from adult AML in Fig. 5C is unclear
(3) Grammar mistakes, including cmalignant in L198, elapse-associated in L216, imune in L229, by in L48, were in L520, Conict-of-interest in L534.

Reviewer #2 (Comments to the Authors (Required)):

The Manuscript from Cui, Lei and colleagues describes data from a single-cell ATAC-seq study involving the analysis of 177,500 cells from 16 pediatric AML patients across three major molecular subtypes (t(8;21), inv(16), FLT3-ITD), comparing relapse-prone (RPS) versus non-relapsed (NRPS) patients. The major finding of this study is that relapse-associated epigenetic signatures are already present at diagnosis, independent of molecular subtype. The signatures are characterized by heightened innate immune/inflammatory pathway activation and a core transcriptional network including the AP-1, RUNX1, SPI-1, and ETS factors.

Currently, there are no clear biomarkers, beyond genetic subtype, that predict outcome in pAML. The study provides a comprehensive analysis of an important ATAC-Seq data set and provides some evidence that such genotype-independent prognostic stratification is possible. However, some important concerns need to be addressed.

First, genetic subtype seems to be a much greater determinant of ATCseq signatures that RPS versus NRPS (which is, perhaps, to be expected). However, it is unclear whether the ATCseq data is equally as effective at predicting RPS/NRPS across subtypes. Figure 2B should be shown in a subtype-specific manner. Additionally, statistical analysis for 2D should be performed to see if there significantly more DAPs in RPS versus NRPS samples across genetic subtypes. A central question here is whether the ATCseq is primarily providing resolution in FLT3-ITD pAMLs.

Second, no gene expression analysis is provided to confirm that the ATAC-seq data is predictive of altered gene expression states in these tumors. Moreover, the authors do not provide data, CHIP analysis or RNA/protein analysis, that the AP-1, RUNX1, SPI-1, and ETS transcriptional network is more active or highly expressed in RPS tumors. Some functional confirmation of the ATACseq data should be provide.

Third, the Kaplan/Meier data from figure 5 should be divided into patient genetic subtypes. Specifically, is the regulatory signature predictive of patient response because it predicts genetic subtype (FLT-ITD, for example). It would be important to show that within a genetic subtype, that the signature is still predictive.

Finally, the statement that "early epigenetic priming of innate immune and inflammatory programs establishes a relapse-permissive state in pAML" is not supported by the data in the paper. While the analysis suggests that immune and inflammatory genes may be expressed differently in RPS tumors, this work does not include any functional validation of this hypothesis or analysis of the inflammatory state of pre-treatment pAML.

Reviewer #3 (Comments to the Authors (Required)):

This manuscript by Cui et al. presents a single-cell chromatin accessibility analysis of pediatric AML samples to investigate whether relapse-associated epigenetic programs are already present at diagnosis. Using a dataset of 177,500 cells from 16 patients, the authors identify subtype-independent chromatin accessibility differences between relapse-prone (RPS) and non-relapse (NRPS) cases, characterized by enrichment of innate immune and inflammatory pathways, expansion of primitive progenitor populations, and activation of a transcription factor network centered on AP-1, RUNX1, and SPI1.

The study addresses an important and timely question and is supported by a technically sound dataset. The results are generally consistent and biologically plausible, and the integration with clinical datasets adds value. Overall, the work is suitable for publication. However, the manuscript in its current form requires moderate revision, primarily to improve clarity, reduce redundancy, and align the interpretation more closely with the data.

Major comments
• The manuscript requires some language editing before it can be considered for publication. Through the text, there are frequent grammatical errors, typographical mistakes, and too long sentences that often combine multiple ideas and make it difficult to follow. Examples of mistakes are:
1) Line 48: by by recurrent
2) Line 173: alterationsalready
3) Line 229: imune/inflammatory
4) Line 216: elapse-associated epigenetic
• In several instances, the manuscript adopts causal language that is not fully supported by the data. For example, statements suggesting that epigenetic changes "establish" relapse susceptibility are based on correlative observations from cross-sectional data. Given the absence of functional validation, these conclusions should be interpreted more cautiously. Therefore, statements should be revised to reflect association rather than causation. For examples:
1) Lines 34-36: "Collectively, these findings demonstrate that early epigenetic priming of innate immune and inflammatory programs establishes a relapse-permissive state in pAML", should be rephased like "Collectively, these findings demonstrate that early epigenetic priming of innate immune and inflammatory programs is associated with a relapse-prone state."
2) Lines 309-312: "Collectively, these findings define a core relapse-associated transcriptional network, whose coordinated activity establishes and maintains the immune/inflammatory chromatin state characteristic of relapse-prone AML, ultimately contributing to adverse clinical outcomes", should be revise as "Collectively, these findings define a core relapse-associated transcriptional network, whose coordinated activity correlates with the immune/inflammatory chromatin state characteristic of relapse-prone AML and may ultimately contribute to adverse clinical outcomes."
3) Lines 333-336: "These findings indicate that relapse susceptibility in pAML is not merely a consequence of treatment-induced selection, but instead reflects early epigenetic priming that establishes a permissive chromatin landscape prior to therapy", should be rephased like "These findings indicate that relapse susceptibility in pAML is not merely a consequence of treatment-induced selection, but may be associated with early epigenetic priming and a permissive chromatin landscape present prior to therapy".

• The Discussion provides a comprehensive interpretation but partially overlaps with the Results and repeats several key observations with minimal variation. In addition, the limitations section could be strengthened. In particular, the manuscripts

should more clearly acknowledge the cross-sectional nature of the data and the lack of functional validation of the proposed regulatory network. The authors should also clearly distinguish speculation from data-supported conclusions.

• Terminology should be standardized throughout the manuscript. Examples include:

1) "non-relapsed" vs. "non-relapse" (the former is preferred).

2) RPS used to refer to both "relapse" and "relapse-prone", which are conceptually distinct as one refers to outcome, the other implies risk/prediction

3) Inconsistent capitalization (NeP vs. Nep; MeP vs. MEP). Standard nomenclature (e. g., NeP-like, MEP-like) should be used consistently.

• The term "LSC6" is introduced in the Results/Figure 2 without prior definition. As this is not a universally familiar metric, the authors should define it at first mention (e.g., as a six-gene leukemia stem cell gene signature score) and briefly indicate how it is derived, with appropriate citation.

• Figure citations are not consistently formatted throughout the manuscript. For example, panel references are variably written as "Fig. 2A-B", "Fig. 4A and B", "Fig. 4C; Fig. S5" and "Fig. 2D and Fig. S3B". The authors should standardize figure citation style (e.g., consistent use of hyphens for ranges and semicolons for separating figures) to improve clarity and presentation.

Minor comments

• Figure legends should be revised for clarity and consistency. In particular, there is redundancy between all figure descriptions in the Results section and the dedicated Figure legends section, and panel labeling (e.g., Figure 1 panels are labeled a-e in the Results but f-j in the legends, despite referring to the same figure).

• Some inconsistencies are present in the reporting of statistical analyses. In particular, thresholds for differential accessibility (e.g., log2 fold-change cutoffs of 1.5 vs. 0.1) differ between the Methods and figure legends, and statistical tests and multiple testing corrections are not always described consistently across sections. Thie should be clarified and standardized to improve reproducibility and clarity.

• The Methods section contains several minor grammatical and formatting issues, including subject-verb agreement errors and missing spaces. Examples include:

1) Line 418: "Standard induction therapy were administered to all patients". This should be revised to "Standard induction therapy was administered to all patients", as "induction therapy" is singular. Additionally, the sentence should begin after a full stop rather than following a comma.

2) Line 426: "Quality control(QC) metrics were applied", missing space after "control".

3) Line 427: "criteria:(i) fewer than 1,000", missing space after colon.

4) Line 475: "false discovery rate FDR {less than or equal to} 0.05", missing parentheses around FDR.

5) Line 476: "motifs enrichment", it should be "motif enrichment".

Response to reviewers' comments

We sincerely thank the Editor and reviewers for your thoughtful evaluation of our manuscript entitled **"Single-cell chromatin profiling reveals early relapse-associated epigenetic priming in pediatric acute myeloid leukemia"** and for providing us with the opportunity to submit a revised version. We are grateful for the insightful comments and suggestions, which have helped us substantially improve the clarity, rigor, and interpretation of the manuscript.

In the revised manuscript, we have carefully addressed all comments raised by the reviewers. The major revisions are summarized as follows: (i) incorporating additional subtype-level analyses for primitive cell proportions, differentially accessible peaks, and survival associations of the key TF signature; (ii) clarifying and further supporting malignant versus healthy-like cell assignment; (iii) adding RNA-seq-based analyses using the TARGET pAML cohort to provide transcriptomic support for the TF regulatory network; (iv) tempering the main claims and strengthening the Discussion by expanding the study limitations and clinical implications; and (v) improving language, terminology consistency, figure citation style, statistical reporting, and figure legends throughout the manuscript.

Below, we provide detailed point-by-point responses to each comment. The reviewers' comments are shown in black, and our responses are shown in blue. All relevant revisions have been highlighted in the revised manuscript.

Reviewer comments:

Reviewer #1 (Comments to the Author):

In this manuscript, the authors investigate early relapse-associated epigenetic features in pediatric acute myeloid leukemia (pAML). This is a very interesting and important question in pAML, especially due to its potential role in predicting relapse. This study uses data mining to investigate these claims and attempts to connect relapse to chromatin accessibility signatures at diagnosis, including enrichment of innate immune and inflammatory pathways, expansion of stem cell-like regulatory programs, and increased accessibility of motifs associated with AP-1, RUNX1, SPI1 and ETS transcription factors. However, some of the conclusions in this manuscript overlap with a previously published Blood paper (Cui et al. 2025), resulting in limited novelty. Without functional validation and larger datasets, some conclusions are overstated. The following points should be addressed prior to publication.

Major comment 1: This manuscript appears to be a re-analysis of the authors' previously published pAML mtscATAC-seq dataset. While the re-analysis of existing data is valuable, the manuscript should clearly explain how it differs from the prior paper. Otherwise, the novelty is limited.

Response: We thank the reviewer for raising this important point. We have substantially revised the Introduction to explicitly distinguish the current study from our prior publication (revised manuscript, Introduction, lines 89–94).

Although the present study builds upon the single-cell chromatin accessibility atlas generated in our previous work, the biological question, patient stratification, analytical framework, and conclusions are distinct. The prior *Blood* study primarily focused on characterizing the epigenetic and clonal architecture of pAML progression, emphasizing subtype heterogeneity, mtDNA-based clonal evolution, and chromatin remodeling between diagnosis and relapse. In contrast, the present manuscript addresses a distinct diagnosis-stage question that was not systematically examined in our previous study: whether patients who subsequently relapse already exhibit

relapse-associated chromatin accessibility programs at initial diagnosis, before therapeutic intervention.

To address this question, we re-stratified diagnostic samples according to long-term clinical outcome into relapse-prone samples (RPS) and non-relapsed samples (NRPS), and performed subtype-aware comparisons to identify pre-existing epigenetic features associated with subsequent relapse. Using this outcome-oriented analytical framework, we identified candidate relapse-associated chromatin accessibility programs detectable at diagnosis, including enhanced innate immune and inflammatory accessibility signatures, enrichment of HSC/MPP-like leukemic progenitors, subtype-resolved RPS-NRPS differentially accessible peak patterns, and a candidate AP-1/RUNX1/SPI1/ETS/BACH regulatory network. We further evaluated the clinical relevance of this regulatory network using independent AML expression cohorts.

We believe these revisions clarify that the current manuscript represents a focused, outcome-oriented extension of the prior pAML atlas, rather than a repetition of the previous study. The novelty of the present study lies in identifying diagnosis-stage relapse-associated epigenetic features and regulatory networks that were not systematically analyzed in our previous publication.

Major comment 2: This manuscript claims the relapse-associated signature is independent of molecular subtype. Considering the small sample size, it is not enough to strongly claim subtype independence.

Response: We thank the reviewer for this important and constructive comment. We agree that the phrase “independent of molecular subtype” was too strong. We have therefore revised the manuscript to avoid this

overstatement. Specifically, we replaced such wording with more cautious statements, including “observed across multiple molecular subtypes” and “not solely attributable to molecular subtype” where appropriate. These revisions have been made in the Abstract, Introduction, Results, and Discussion (revised manuscript, Abstract, lines 26–28, 33–34; Introduction, lines 99–101; Results, lines 139–144, 192–194 and 335–338; Discussion, lines 367–370).

To further address this concern, we performed additional subtype-aware analyses and added new supporting figures. First, we revised Figure 2B to show the comparison of primitive cell proportions between RPS and NRPS samples with individual samples overlaid and colored by molecular subtype (Response Figure 1A). This visualization allows the RPS–NRPS difference to be interpreted in the context of subtype composition (revised manuscript, Results, lines 192–194). Because t(8;21) represented the largest subtype in our cohort, we also performed a subtype-specific analysis of primitive cell proportions within t(8;21) samples (Response Figure 1B). This analysis showed a similar trend of increased primitive cell proportions in RPS samples and is included in the response file as supporting evidence.

Second, we expanded the subtype-resolved analysis of differentially accessible peaks (DAPs). We added new supplementary panels showing the total numbers of RPS-associated and NRPS-associated DAPs across the three molecular subtypes, as well as a paired comparison of RPS- versus NRPS-associated DAPs across matched subtype–cell type pairs (Response Figure 1C–D). These analyses are now included as Figure S3C and Figure S3D, respectively (revised manuscript, Results, lines 213–218). Across matched subtype–cell type pairs, RPS-associated DAPs were significantly more abundant than NRPS-associated DAPs (paired Wilcoxon signed-rank test, p = 1.35e-05), supporting the presence of relapse-associated chromatin accessibility differences across multiple molecular subtypes.

Third, to evaluate whether the TF signature in Figure 5 was driven by a single molecular subtype, we performed subtype-stratified Kaplan–Meier survival analyses in the TARGET pAML cohort (Response Figure 1E–G). These results are now included in Figure S6A–C (revised manuscript, Results, lines 335–338). The key TF expression signature was associated with inferior survival within *FLT3*-ITD, t(8;21), and inv(16) subgroups, supporting the clinical relevance of this regulatory program across multiple molecular contexts. We have revised the manuscript text accordingly.

Together, these revisions support the conclusion that, although genetic subtype strongly shapes the overall chromatin landscape of pAML, relapse-associated epigenetic and regulatory features are detectable across multiple analyzed subtypes and are not solely driven by a single molecular subtype.

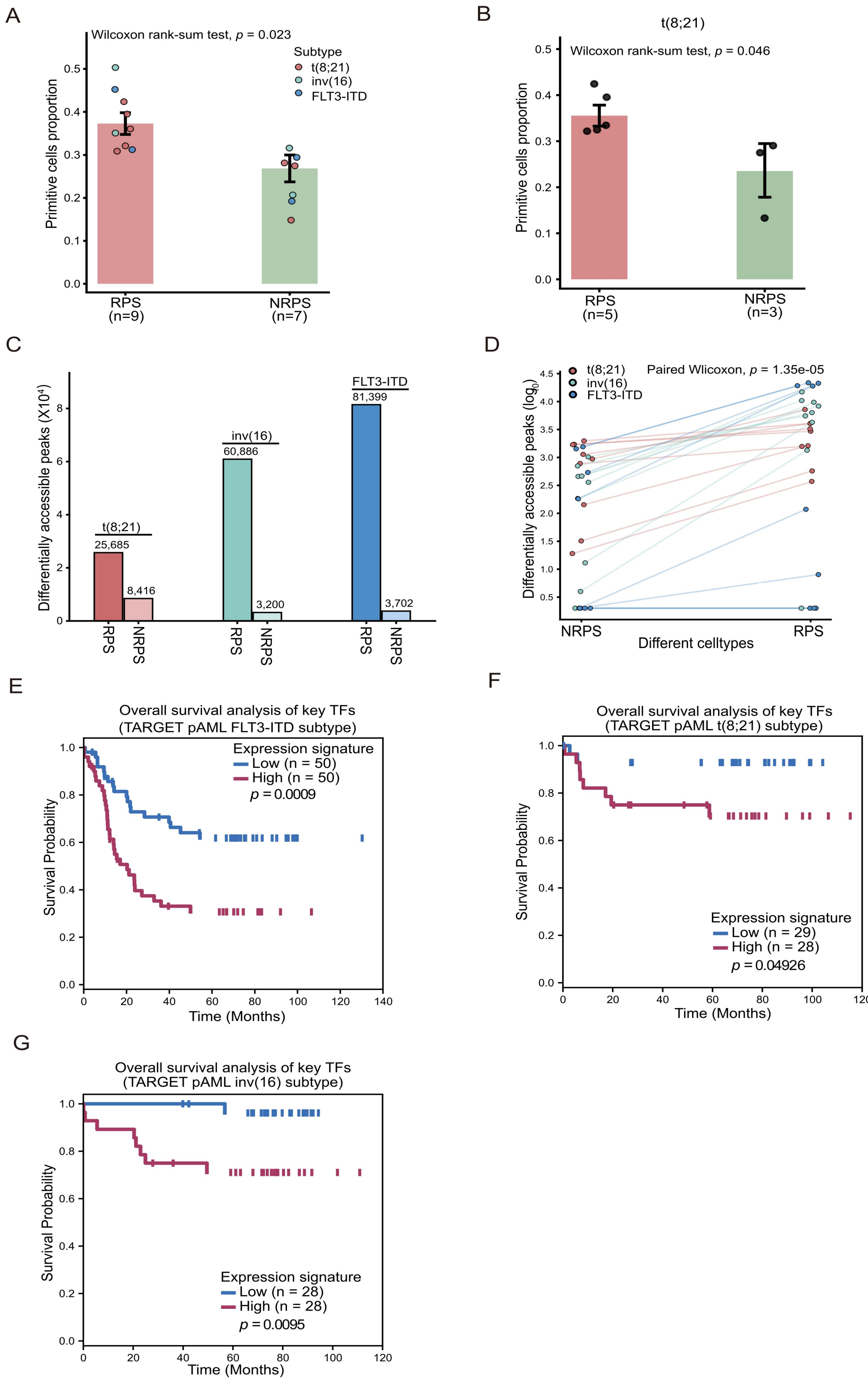
A
Wilcoxon rank-sum test, p = 0.023
Subtype
t(8;21)
inv(16)
FLT3-ITD
Primitive cells proportion
RPS (n=9)
NRPS (n=7)
B
t(8;21)
Wilcoxon rank-sum test, p = 0.046
Primitive cells proportion
RPS (n=5)
NRPS (n=3)
C
Differentially accessible peaks (X10⁴)
t(8;21)
25,685
8,416
inv(16)
60,886
3,200
FLT3-ITD
81,399
3,702
RPS
NRPS
D
t(8;21)
inv(16)
FLT3-ITD
Paired Wlicoxon, p = 1.35e-05
Differentially accessible peaks (log10)
NRPS
RPS
Different celltypes
E
Overall survival analysis of key TFs
(TARGET pAML FLT3-ITD subtype)
Expression signature
Low (n = 50)
High (n = 50)
p = 0.0009
Survival Probability
Time (Months)
F
Overall survival analysis of key TFs
(TARGET pAML t(8;21) subtype)
Expression signature
Low (n = 29)
High (n = 28)
p = 0.04926
Survival Probability
Time (Months)
G
Overall survival analysis of key TFs
(TARGET pAML inv(16) subtype)
Expression signature
Low (n = 28)
High (n = 28)
p = 0.0095
Survival Probability
Time (Months)

Response Figure 1. Subtype-aware analyses supporting relapse-associated cellular composition, chromatin accessibility, and TF-signature features across three molecular subtypes.

A. Revised Figure 2B: Comparison of primitive cell proportions between RPS and NRPS samples, with individual samples overlaid and colored by molecular subtype.

B. t(8;21)-specific comparison of primitive cell proportions between RPS and NRPS samples.

C. Revised Figure S3C: Total numbers of DAPs more accessible in RPS or NRPS samples within each molecular subtype.

D. Revised Figure S3D: Paired comparison of RPS- versus NRPS-associated DAP counts across matched subtype–cell type pairs.

E–G. Revised Figure S6A–C: Subtype-stratified Kaplan–Meier survival analyses of the key TF expression signature in *FLT3*-ITD, t(8;21), and inv(16) subgroups of the TARGET pAML cohort.

Major comment 3: The correct malignant cell assignment is important for downstream analysis. The authors refer to their previous pAML atlas for reference-based annotation, but in this manuscript, it is still unclear how malignant cells were distinguished from normal cells, which may potentially contribute to the observed difference in chromatin accessibility.

Response: We thank the reviewer for this important suggestion. We agree that clear description and validation of malignant cell assignment are essential for interpreting downstream chromatin accessibility differences. We have therefore expanded both the Results and Methods sections to describe our malignant cell classification strategy more explicitly.

Briefly, malignant cells were identified using an integrated strategy established in our previous pAML mtscATAC-seq atlas. This strategy incorporates multiple layers of evidence, including leukemia-associated genomic variants, copy-number signatures, AML-related chromatin accessibility patterns, and concordance with flow cytometric blast percentages (revised manuscript, Results, lines 123–127; Response Figure 2A–F). In the current manuscript, we now describe this classification framework more clearly and explain that downstream RPS versus NRPS chromatin accessibility analyses were restricted to confidently assigned malignant cells.

As a new validation for this specific cohort, we performed ratio-of-observed-to-expected (Ro/e) analyses comparing classified disease cells and healthy-like cells with healthy control hematopoietic cells across annotated cell types. These analyses showed that healthy-like cells exhibited a distribution comparable to healthy control hematopoietic populations, whereas classified disease cells were preferentially enriched in primitive and progenitor-like compartments (revised manuscript, Results, lines 127–133; revised Figure S2A–B; Response Figure 2G–H). These results support the reliability of malignant cell assignment in the current cohort and reduce the possibility that the observed RPS–NRPS chromatin accessibility differences are driven by contaminating normal hematopoietic cells.

We have also incorporated a concise description of the malignant cell assignment strategy into the Methods section. Specifically, single-cell chromatin accessibility profiles from pAML samples were projected onto a pediatric healthy hematopoietic reference map to distinguish disease cells from healthy-like hematopoietic cells. Cells that deviated from normal hematopoietic chromatin states and clustered within leukemia-associated regions were classified as disease cells, whereas cells mapping to normal hematopoietic compartments were classified as healthy-like cells. We further

clarified that additional validation of malignant cell assignment, including leukemia-associated genomic signals, copy-number alterations, AML-related chromatin signatures, and concordance with clinical blast percentages, was performed in our previous pAML atlas (revised manuscript, Methods, lines 479–493).

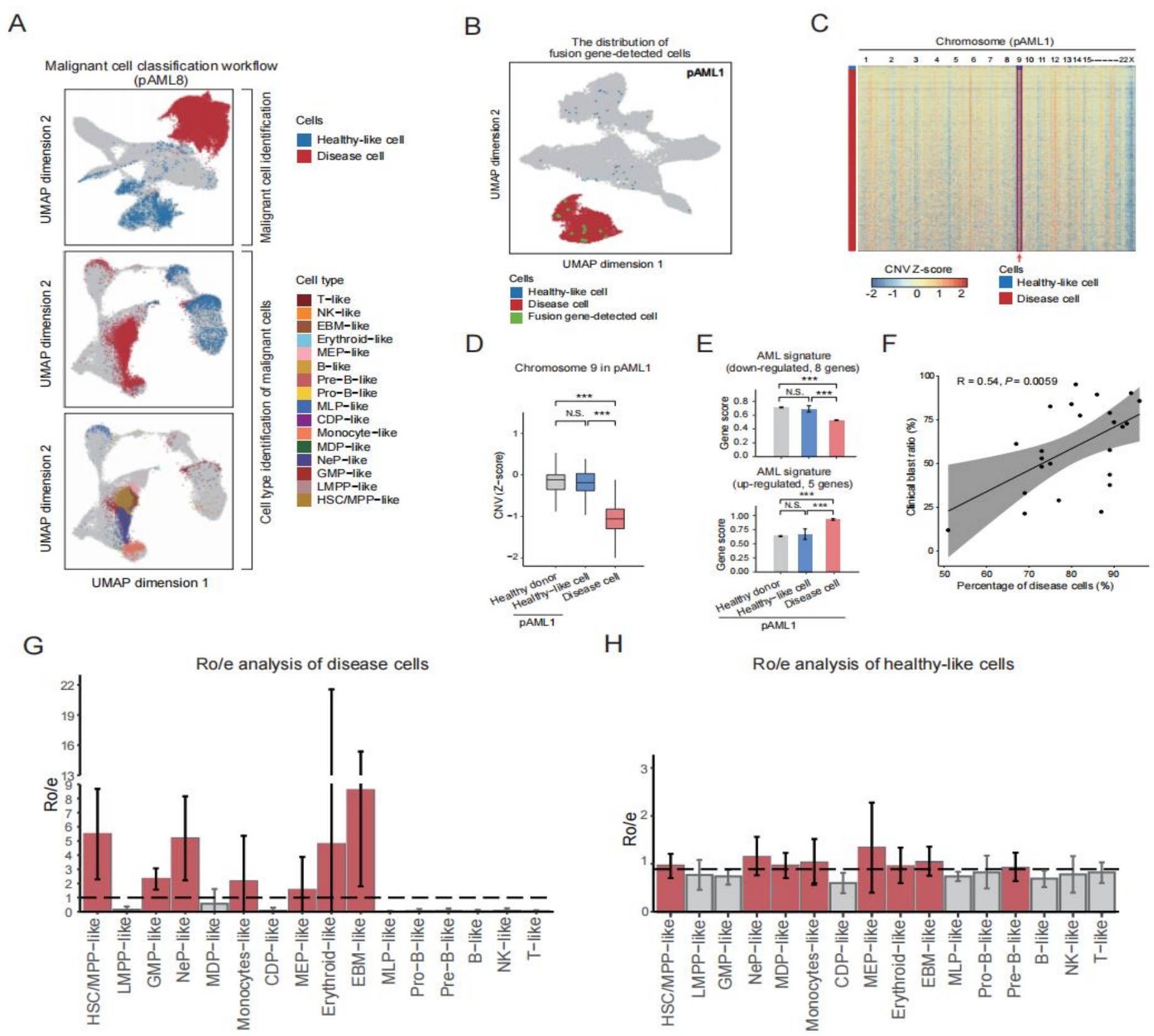

Response Figure 2. Validation of malignant cell assignment in the pAML mtscATAC-seq dataset.

A–F. Supporting evidence from the previously published pAML mtscATAC-seq atlas in Blood. These panels summarize the original malignant cell classification workflow and validation analyses, including disease-cell and healthy-like cell assignment, fusion gene-detected cells, copy-number alteration signals, AML-related chromatin accessibility signatures, and

concordance between classified disease-cell proportions and clinical blast percentages.

G. Revised Figure S2A: Ro/e analysis comparing the distribution of classified disease cells with healthy control hematopoietic cells across annotated cell types.

H. Revised Figure S2B: Ro/e analysis comparing the distribution of healthy-like cells from pAML samples with healthy control hematopoietic cells across annotated cell types.

Major comment 4: In the chromatin accessibility analysis beginning at line 208, the authors conclude that the observed differences reflect cell-intrinsic regulatory alterations rather than compositional effects. This conclusion is too strong. Although the reported correlation is not statistically significant, the limited sample size and moderate positive trend do not exclude the possibility that cell abundance contributes to the number of differentially accessible regions. The authors should explore other normalization methods.

Response: We thank the reviewer for this important and constructive comment. We agree that our previous interpretation was too strong. To address this concern, we performed three additional analyses and revised the presentation of the original analysis. First, we revised the original correlation analysis between cell number and DAP counts by presenting both variables on a log10-transformed scale, which is more appropriate for variables spanning different orders of magnitude (Response Figure 3A). This analysis is now shown as Figure S3E in the revised manuscript (revised manuscript, Results, lines 218–222). Although the association remained statistically non-significant, we now interpret this result cautiously and do not use it as definitive evidence for cell-intrinsic regulation.

Second, we performed an exploratory downsampling analysis to reduce the potential influence of unequal cell numbers across malignant cell types (Response Figure 3B). Briefly, for each malignant cell type, we randomly downsampled comparable numbers of cells between RPS and NRPS groups where possible and repeated this procedure 100 times. Differentially accessible peaks were then recalculated after downsampling, and DAP counts were summarized across repeated iterations. This analysis showed that several progenitor-like populations, including HSC/MPP-like, NeP-like, EBM-like, and MEP-like cells, still exhibited substantial numbers of RPS–NRPS DAPs after cell-number normalization. These results suggest that the observed chromatin accessibility differences are not solely explained by cell-number imbalance, although they do not exclude a contribution from cell abundance.

Third, we further compared malignant cell-type composition between RPS and NRPS samples (Response Figure 3C–D). This analysis showed that DAP burden was not simply proportional to cell-type abundance. For example, some relatively low-abundance progenitor-like populations, such as EBM-like and MEP-like cells, still showed substantial DAP numbers after downsampling, whereas some more abundant populations did not show proportionally higher DAP counts.

Together, these additional analyses support a more cautious interpretation: differences in cell abundance are unlikely to fully account for the observed RPS–NRPS DAP patterns, although a partial contribution from cell-type composition cannot be excluded. Accordingly, we revised the corresponding manuscript text to state that relapse-associated chromatin accessibility differences are “unlikely to be explained solely by cell-type abundance and may partly reflect cell-intrinsic regulatory alterations,” rather than presenting them as definitive evidence of purely cell-intrinsic regulation.

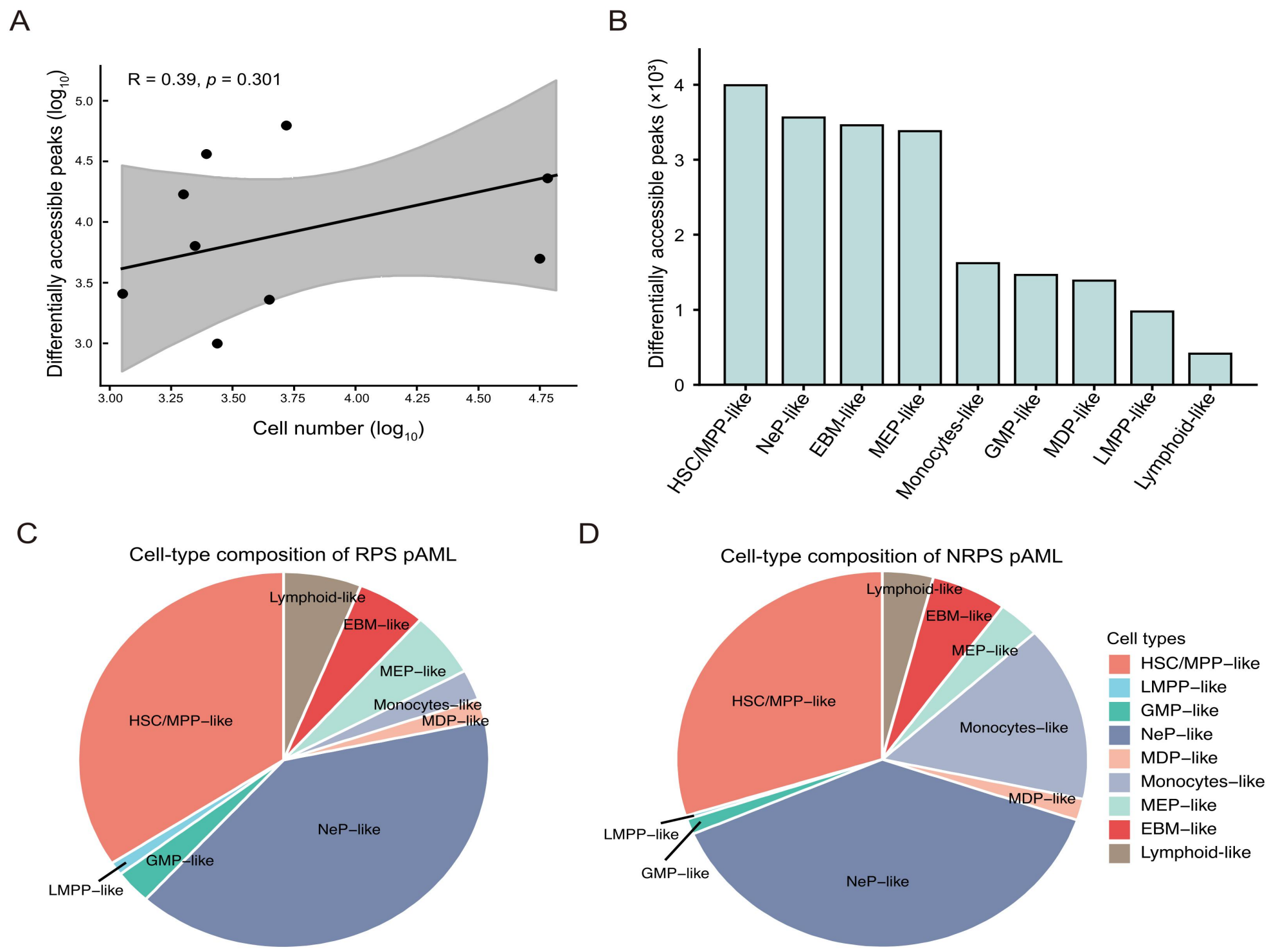

Response Figure 3. Assessment of cell-abundance effects on RPS–NRPS differentially accessible peak patterns.

A. Revised Figure S3E: Scatter plot showing the correlation between the number of sequenced cells and the number of differentially accessible peaks (DAPs) after log10 transformation of both variables. The Spearman correlation coefficient (R), *p* value, and 95% confidence interval for the regression line are shown.

B. Downsampling-based DAP analysis across malignant cell types. For each malignant cell type, comparable numbers of cells were randomly sampled between RPS and NRPS groups where possible, and DAP counts were recalculated across 100 repeated resampling iterations. Bars indicate the mean DAP count across iterations.

C–D. Cell-type composition of RPS (C) and NRPS (D) pAML samples, respectively.

Major comment 5: As written, the study identifies candidate relapse-associated molecular features rather than validated early predictors of relapse. The manuscript should therefore temper the use of "predictors" unless independent validation and multivariable predictive modeling are provided.

Response: We thank the reviewer for this important and constructive comment. We agree that the present study identifies candidate relapse-associated molecular features rather than validated early predictors of relapse. We have therefore tempered the language throughout the manuscript to avoid overstatement.

Specifically, prediction-related terms such as “predictors”, “predictive biomarkers” and “relapse prediction” have been replaced, where appropriate, with more cautious associative terminology, including “candidate relapse-associated features”, “candidate biomarkers”, “features associated with subsequent relapse” and “relapse-associated molecular features” (revised manuscript, Abstract, lines 33–38; Introduction, lines 108–110; Results, lines 142–144, 222–225, 331–334 and 342–345; Discussion, lines 410–413 and 437–441). In the Discussion, we now explicitly acknowledge that larger independent cohorts, prospective validation, and multivariable predictive modeling will be required to determine whether these candidate relapse-associated features have clinical predictive value (revised manuscript, Discussion, lines 429–432).

Together, these revisions better distinguish candidate relapse-associated molecular features from validated relapse predictors and more accurately reflect the evidence provided by the present study.

Minor comments

Minor comments 1: Fig. 2D should clarify if the differentially peaks are more open or closed in RPS or NRPS.

Response: We thank the reviewer for this helpful suggestion. We have revised Figure 2D and its legend to explicitly indicate the direction of differential accessibility. Specifically, positive values represent peaks that are more accessible, or more open, in RPS samples, whereas negative values represent peaks that are more accessible, or more open, in NRPS samples. This clarification has been added to the revised Figure 2D legend (revised manuscript, Figure 2D legend, lines 237–240).

Minor comments 2: The relevance of the survival analyses from adult AML in Fig. 5C is unclear.

Response: We thank the reviewer for this helpful comment. We have clarified that the TCGA-LAML cohort was not intended as direct validation of a pediatric AML-specific relapse-associated signature. Rather, it was included as an independent adult AML cohort to assess whether the TF network identified from pAML chromatin accessibility analysis also shows adverse clinical association in a broader AML context (revised manuscript, Results, lines 331–334; Discussion, lines 394–396).

Minor comments 3: Grammar mistakes, including cmalignant in L198, elapse-associated in L216, imune in L229, by in L48, were in L520, Conict-of-interest in L534.

Response: We thank the reviewer for carefully identifying these grammatical and typographical errors. We have corrected all of the listed errors and performed a thorough language check throughout the revised manuscript.

Reviewer #2 (Comments to the Author):

Comment 1: Genetic subtype seems to be a much greater determinant of ATCseq signatures that RPS versus NRPS (which is, perhaps, to be expected). However, it is unclear whether the ATCseq data is equally as effective at predicting RPS/NRPS across subtypes. Figure 2B should be shown in a subtype-specific manner. Additionally, statistical analysis for 2D should be performed to see if there significantly more DAPs in RPS versus NRPS samples across genetic subtypes. A central question here is whether the ATCseq is primarily providing resolution in *FLT3*-ITD pAMLs.

Response: We thank the reviewer for this important and insightful comment. We agree that genetic subtype is a major determinant of the global chromatin accessibility landscape in pAML, as reflected by the subtype-associated clustering patterns in Figure 1C–D. We also agree that it is important to determine whether the observed RPS–NRPS differences are present across molecular subtypes or are primarily driven by *FLT3*-ITD samples. To directly address this concern, we performed three additional subtype-aware analyses and revised the corresponding figures and text.

First, we revised Figure 2B to display individual samples overlaid on the RPS and NRPS comparison and colored by molecular subtype (Response Figure 1A; revised manuscript, Results, lines 192–194; Figure 2B). This allows the difference in primitive cell proportions between RPS and NRPS samples to be evaluated in the context of subtype composition. Because t(8;21) represented the largest molecular subtype in our cohort, we also performed a

t(8;21)-specific comparison of primitive cell proportions as an additional supporting analysis (Response Figure 1B). This analysis showed a similar trend toward increased primitive cell proportions in RPS samples. We interpret this result cautiously because of the limited within-subtype sample size.

Second, we expanded the DAP analysis in a subtype-resolved manner. We added Figure S3C to show the total numbers of DAPs with higher accessibility in RPS samples or NRPS samples within each molecular subtype (Response Figure 1C). This analysis showed that RPS-associated DAPs were observed across t(8;21), inv(16), and *FLT3*-ITD samples, rather than being restricted to *FLT3*-ITD pAML. We further added Figure S3D to statistically compare RPS- versus NRPS-associated DAP counts across matched subtype-cell type pairs (Response Figure 1D). This paired analysis showed that DAPs with higher accessibility in RPS samples were significantly more abundant than DAPs with higher accessibility in NRPS samples across matched subtype-cell type pairs (paired Wilcoxon signed-rank test, p = 1.35e-05; revised manuscript, Results, lines 210–218; Figure S3C–D). These analyses directly address the reviewer's request for statistical assessment of RPS- versus NRPS-associated DAPs across genetic subtypes.

Third, as an additional clinical supporting analysis, we performed subtype-stratified Kaplan–Meier survival analyses of the relapse-associated TF expression signature in the TARGET pAML cohort (Response Figure 1E–G), which are now included as Figure S6A–C. The TF signature was associated with inferior survival within *FLT3*-ITD, t(8;21), and inv(16) subgroups, suggesting that the clinical association of this regulatory program is not confined to *FLT3*-ITD pAML (revised manuscript, Results, lines 335–338; Figure S6A–C).

Together, these analyses support the conclusion that, although genetic subtype strongly shapes the global ATAC-seq landscape, relapse-associated chromatin accessibility and regulatory features are observed across multiple molecular subtypes and are not solely driven by *FLT3*-ITD samples. We have revised the manuscript text accordingly to avoid overstatement and to present these subtype-aware analyses more accurately.

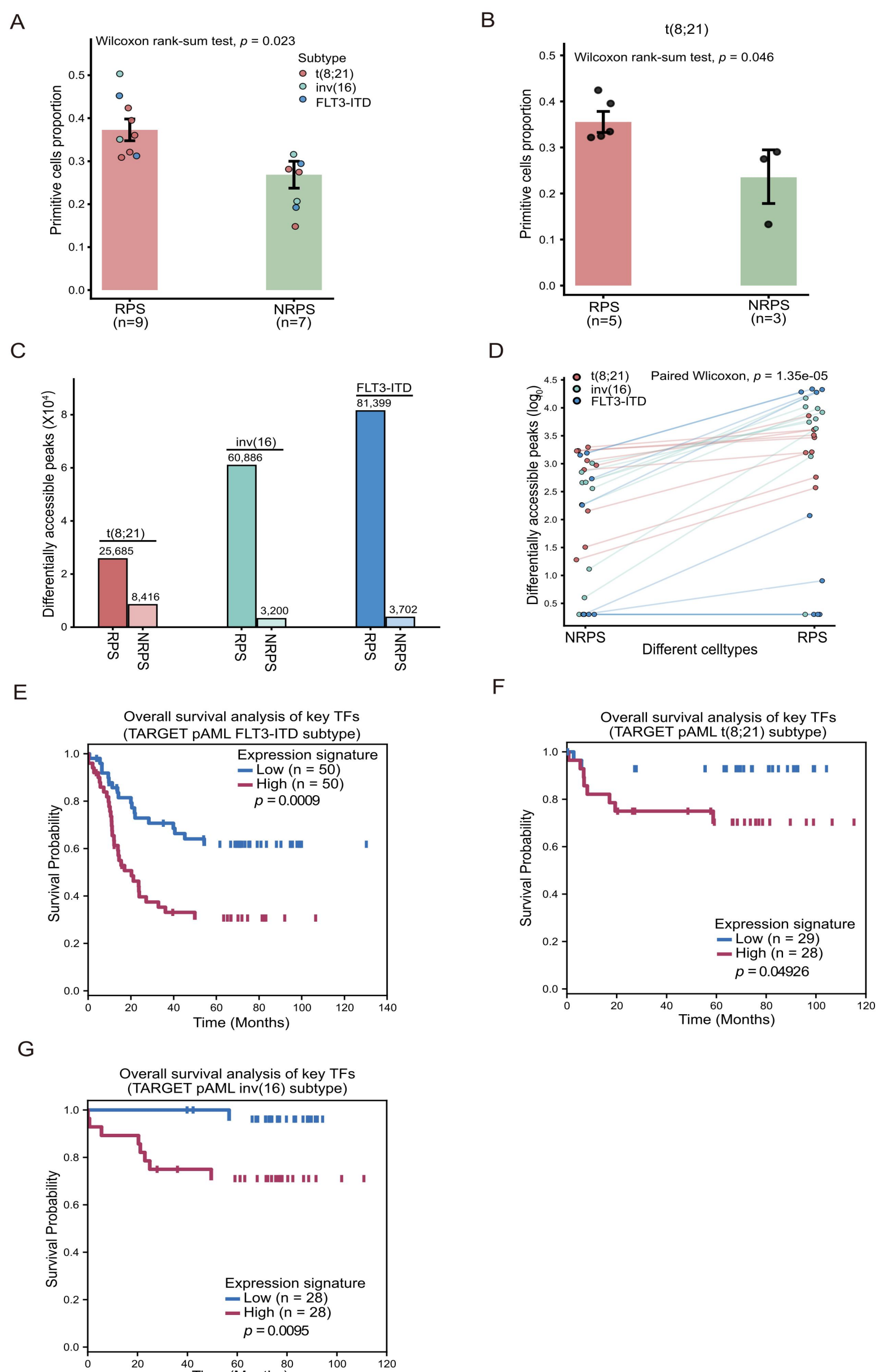

Response Figure 1. Subtype-aware analyses supporting relapse-associated cellular composition, chromatin accessibility, and TF-signature features across three molecular subtypes.

A. Revised Figure 2B: Comparison of primitive cell proportions between RPS and NRPS samples, with individual samples overlaid and colored by molecular subtype.

B. t(8;21)-specific comparison of primitive cell proportions between RPS and NRPS samples.

C. Revised Figure S3C: Total numbers of DAPs more accessible in RPS or NRPS samples within each molecular subtype.

D. Revised Figure S3D: Paired comparison of RPS- versus NRPS-associated DAP counts across matched subtype–cell type pairs.

E–G. Revised Figure S6A–C: Subtype-stratified Kaplan–Meier survival analyses of the key TF expression signature in *FLT3*-ITD, t(8;21), and inv(16) subgroups of the TARGET pAML cohort.

Comment 2: No gene expression analysis is provided to confirm that the ATAC-seq data is predictive of altered gene expression states in these tumors. Moreover, the authors do not provide data, CHIP analysis or RNA/protein analysis, that the AP-1, RUNX1, SPI-1, and ETS transcriptional network is more active or highly expressed in RPS tumors. Some functional confirmation of the ATACseq data should be provide.

Response: We thank the reviewer for this important and constructive comment. We agree that gene expression-level evidence is important for supporting the biological relevance of the ATAC-seq-derived regulatory network. To address this concern, we performed additional transcriptomic analyses using the independent TARGET pediatric AML RNA-seq cohort to evaluate whether the transcription factors identified from our chromatin

accessibility analyses are associated with adverse gene expression states and clinical features.

Specifically, we calculated a TF expression signature score based on the key regulators identified from our ATAC-seq motif enrichment, TF footprinting, and PPI network analyses, including AP-1 family members, RUNX1, SPI1, ETS-related factors, BACH family members, and JDP2. This RNA-based TF signature was significantly higher in high-risk patients than in standard-risk and low-risk patients in the TARGET pAML cohort, and this result has been added as Figure 5D (Response Figure 4A). In addition, deceased patients showed significantly higher TF expression signature scores than surviving patients in the TARGET pAML cohort, and this analysis has been added as Figure S6D (revised manuscript, Results, lines 338-342; Response Figure 4B). We also revised the Methods to describe how the TF expression signature score was calculated from RNA-seq data (revised manuscript, Methods, lines 551–565).

Together with the ATAC-seq motif enrichment, TF footprinting, and PPI network analyses, these RNA-seq results provide orthogonal transcriptomic support that the chromatin-derived TF network is associated with higher clinical risk and adverse survival-related features in pediatric AML. We have revised the Results, figure legends, and Methods to describe these additional analyses. We have also added a statement in the Discussion acknowledging that direct functional validation, including perturbation assays and protein-level validation, will be required in future studies to determine whether this TF network directly regulates relapse-associated chromatin and transcriptional states (revised manuscript, Discussion, lines 429–432).

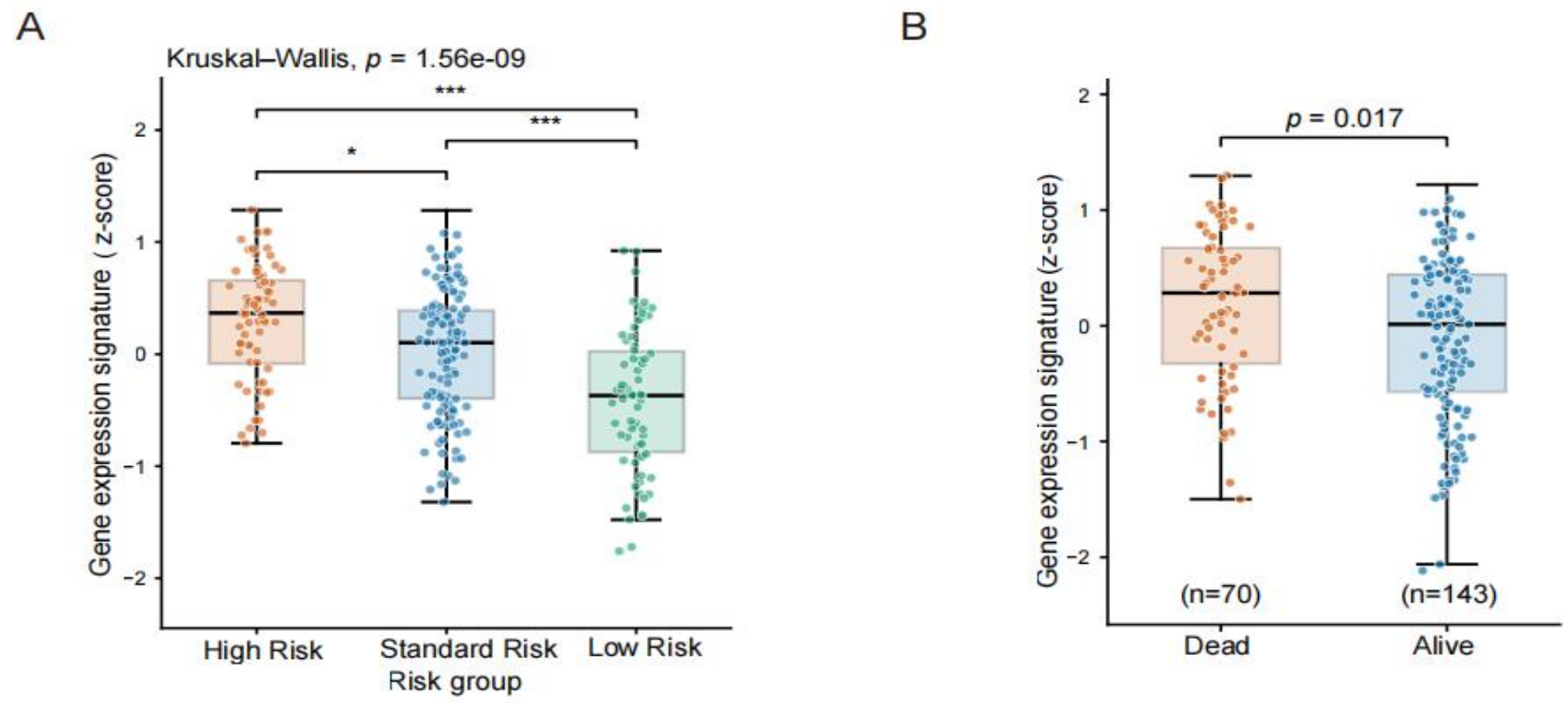

Response Figure 4. Transcriptomic support for the clinical association of the relapse-associated TF expression signature.

A. Revised Figure 5D: Box plot showing the key TF expression signature score across TARGET pAML clinical risk groups. Statistical significance was assessed using the Kruskal–Wallis test, followed by Wilcoxon rank-sum test.

B. Revised Figure S6D: Box plot showing the key TF expression signature score in deceased and surviving patients from the TARGET pAML cohort. Statistical significance was assessed using the Wilcoxon rank-sum test.

Comment 3: Third, the Kaplan/Meier data from figure 5 should be divided into patient genetic subtypes. Specifically, is the regulatory signature predictive of patient response because it predicts genetic subtype (*FLT3*-ITD, for example). It would be important to show that within a genetic subtype, that the signature is still predictive.

Response: We thank the reviewer for this important suggestion. To address this concern, we performed subtype-stratified Kaplan–Meier survival analyses in the TARGET pediatric AML cohort. Within each genetic subtype, patients were stratified into high- and low-signature groups using the median TF

expression signature score of that subtype as the cutoff (Response Figure 1E–G). These analyses are now included as Figure S6A–C. High TF signature scores was associated with inferior overall survival within the *FLT3*-ITD subtype, as well as within the t(8;21) and inv(16) subtypes. These results indicate that the adverse survival association of the relapse-associated TF signature is not restricted to a specific pAML subtype.

We have revised the Results section to describe these subtype-stratified survival analyses and added Figure S6A–C together with the corresponding figure legend (revised manuscript, Results, lines 335–338). These additions clarify that the regulatory signature remains associated with prognosis within individual molecular subtypes, supporting its clinical relevance across distinct genetic contexts in pAML.

Comment 4: Finally, the statement that "early epigenetic priming of innate immune and inflammatory programs establishes a relapse-permissive state in pAML" is not supported by the data in the paper. While the analysis suggests that immune and inflammatory genes may be expressed differently in RPS tumors, this work does not include any functional validation of this hypothesis or analysis of the inflammatory state of pre-treatment pAML.

Response: We thank the reviewer for this important comment. We fully agree that our previous wording implied a causal relationship that cannot be established by the current data. We have therefore revised the manuscript to avoid causal overstatement. Specifically, we now describe these findings more cautiously as immune- and inflammation-associated chromatin accessibility programs that are observed in relapse-prone pAML samples at diagnosis and may be linked to relapse susceptibility (revised manuscript, Abstract, lines 26–28; Results, lines 265–269; Discussion, lines 370–373, 437–441).

We also clarified the scope of the analysis. All pAML samples analyzed in this study were obtained at diagnosis before chemotherapy; therefore, the immune and inflammatory features described here reflect diagnosis-stage, pre-treatment chromatin accessibility differences between RPS and NRPS samples. Accordingly, we now refer to these features as immune- and inflammation-associated chromatin accessibility signatures, rather than as a functionally validated inflammatory state.

As additional supportive evidence, we performed RNA-seq-based analyses using the independent TARGET pediatric AML cohort. We calculated a TF expression signature score based on the key regulators identified from ATAC-seq motif enrichment, footprinting, and PPI network analyses. This signature was significantly elevated in high-risk pediatric AML patients compared with standard-risk and low-risk patients and was also higher in deceased patients than in surviving patients (Response Figure 4). These analyses have been added as Figure 5D and Figure S6D, respectively (revised manuscript, Results, lines 338–342; Figure 5D and Figure S6D). Together with the ATAC-seq motif and footprinting results, these data provide orthogonal transcriptomic support that the chromatin-derived TF network is associated with adverse clinical features in pAML.

Importantly, we acknowledge that these transcriptomic analyses do not replace direct functional validation experiments. Accordingly, we revised the Discussion and limitations section to state that future studies integrating longitudinal multi-omics profiling, protein-level assays, and functional perturbation will be required to determine whether these regulators causally contribute to inflammatory chromatin remodeling and relapse susceptibility in pAML (revised manuscript, Discussion, lines 425–432). Together, these revisions more accurately reflect the scope of the data.

Reviewer #3 (Comments to the Author):

Major comment 1: The manuscript requires some language editing before it can be considered for publication. Through the text, there are frequent grammatical errors, typographical mistakes, and too long sentences that often combine multiple ideas and make it difficult to follow. Examples of mistakes are:

1) Line 48: by by recurrent
2) Line 173: alterationsalready
3) Line 229: imune/inflammatory
4) Line 216: elapse-associated epigenetic

Response: We thank the reviewer for carefully pointing out these language issues. We have revised the manuscript throughout to improve grammar, spelling, sentence structure, and overall readability. We have also shortened, separated, or restructured several overly long sentences that previously combined multiple ideas, particularly in the Abstract, Introduction, Results, and Discussion sections. We believe that these edits have improved the readability, precision, and overall presentation of the manuscript.

Major comment 2: In several instances, the manuscript adopts causal language that is not fully supported by the data. For example, statements suggesting that epigenetic changes "establish" relapse susceptibility are based on correlative observations from cross-sectional data. Given the absence of functional validation, these conclusions should be interpreted more cautiously. Therefore, statements should be revised to reflect association rather than causation. For examples:

1) Lines 34-36: "Collectively, these findings demonstrate that early epigenetic priming of innate immune and inflammatory programs establishes a

relapse-permissive state in pAML", should be rephased like "Collectively, these findings demonstrate that early epigenetic priming of innate immune and inflammatory programs is associated with a relapse-prone state."

2) Lines 309-312: "Collectively, these findings define a core relapse-associated transcriptional network, whose coordinated activity establishes and maintains the immune/inflammatory chromatin state characteristic of relapse-prone AML, ultimately contributing to adverse clinical outcomes", should be revise as "Collectively, these findings define a core relapse-associated transcriptional network, whose coordinated activity correlates with the immune/inflammatory chromatin state characteristic of relapse-prone AML and may ultimately contribute to adverse clinical outcomes."

3) Lines 333-336: "These findings indicate that relapse susceptibility in pAML is not merely a consequence of treatment-induced selection, but instead reflects early epigenetic priming that establishes a permissive chromatin landscape prior to therapy", should be rephased like "These findings indicate that relapse susceptibility in pAML is not merely a consequence of treatment-induced selection, but may be associated with early epigenetic priming and a permissive chromatin landscape present prior to therapy".

Response: We thank the reviewer for this careful and constructive comment. We agree completely and have systematically revised the manuscript to eliminate unsupported causal claims. The specific examples pointed out by the reviewer have been revised as suggested.

Specifically, we revised the statement in the Abstract that “early epigenetic priming of innate immune and inflammatory programs establishes a relapse-permissive state in pAML” to indicate that these programs are associated with a relapse-prone state in pAML (revised manuscript, Abstract, lines 34–38). In the Results section, we revised the statement that the relapse-associated transcriptional network “establishes and maintains” the

immune/inflammatory chromatin state to state that this network is associated with the immune/inflammatory chromatin state characteristic of RPS pAML and may be linked to adverse clinical outcomes (revised manuscript, Results, lines 342–345). In the Discussion, we revised the statement that relapse susceptibility “reflects early epigenetic priming that establishes a permissive chromatin landscape” to a more cautious interpretation, indicating that relapse susceptibility may be associated with pre-existing epigenetic differences detectable at diagnosis, rather than being explained solely by treatment-induced selection (revised manuscript, Discussion, lines 370–373).

In addition to these specific revisions, we reviewed the manuscript for similar overstatements and revised causal wording where appropriate. This cautious interpretation is now maintained throughout the manuscript.

Major comment 3: The Discussion provides a comprehensive interpretation but partially overlaps with the Results and repeats several key observations with minimal variation. In addition, the limitations section could be strengthened. In particular, the manuscripts should more clearly acknowledge the cross-sectional nature of the data and the lack of functional validation of the proposed regulatory network. The authors should also clearly distinguish speculation from data-supported conclusions.

Response: We thank the reviewer for this constructive comment. We agree that the original Discussion partially overlapped with the Results and that some interpretations should be presented more cautiously. We have therefore revised the Discussion to reduce repetition of observations and to focus more clearly on interpretation and biological implications. We also revised causal or speculative wording where appropriate (revised manuscript, Discussion, lines 367–378, 394–396, 410–413,437–441).

We further strengthened the limitations section. We now explicitly acknowledge the cross-sectional nature of the data, the limited sample size, the lack of protein-level and functional validation, and the need for longitudinal studies to establish causality and track the evolution of these programs over time (revised manuscript, Discussion, lines 424–432).

Major comment 4: Terminology should be standardized throughout the manuscript. Examples include:

1) "non-relapsed" vs. "non-relapse" (the former is preferred).

2) RPS used to refer to both "relapse" and "relapse-prone", which are conceptually distinct as one refers to outcome, the other implies risk/prediction

3) Inconsistent capitalization (NeP vs. Nep; MeP vs. MEP). Standard nomenclature (e. g., NeP-like, MEP-like) should be used consistently.

Response: We thank the reviewer for this helpful suggestion. We have standardized terminology throughout the revised manuscript and figure legends. We now use “non-relapsed” consistently when referring to patients or samples without subsequent relapse. We have also clarified the definitions of the clinical outcome groups at their first appearance and in the Methods section. Specifically, RPS is now defined as relapse samples, referring to diagnostic samples from patients who subsequently experienced relapse within three years after remission, whereas NRPS is defined as non-relapsed samples, referring to diagnostic samples from patients who remained in sustained remission for at least five years (revised manuscript, Methods, lines 453–456). In addition, we standardized cell-type nomenclature throughout the manuscript and figure legends, including consistent use of abbreviations such as NeP-like, MEP-like, and EBM-like. We also checked capitalization and abbreviation formatting throughout the revised manuscript.

Major comment 5: The term "LSC6" is introduced in the Results/Figure 2 without prior definition. As this is not a universally familiar metric, the authors should define it at first mention (e.g., as a six-gene leukemia stem cell gene signature score) and briefly indicate how it is derived, with appropriate citation.

Response: We thank the reviewer for this helpful comment. We have revised the Results section to define LSC6 at its first mention. Specifically, LSC6 is now described as a previously established six-gene leukemia stem cell (LSC) expression signature developed to quantify stemness-associated transcriptional programs and identify high-risk pediatric AML patients, with citation to Elsayed et al. (2020) (revised manuscript, Results, lines 195–198).

Major comment 6: Figure citations are not consistently formatted throughout the manuscript. For example, panel references are variably written as "Fig. 2A-B", "Fig. 4A and B", "Fig. 4C; Fig. S5" and "Fig. 2D and Fig. S3B". The authors should standardize figure citation style (e.g., consistent use of hyphens for ranges and semicolons for separating figures) to improve clarity and presentation.

Response: We thank the reviewer for this helpful suggestion. We have standardized figure citation formatting throughout the revised manuscript and figure legends to improve clarity and consistency.

Specifically, panel ranges within the same figure are now consistently cited using en dashes, such as “Fig. 2A–B” and “Fig. S4A–C,” whereas references to different figures are separated by semicolons, such as “Fig. 2D; Fig. S3B” and “Fig. 4C; Fig. S5.” We also reviewed the manuscript to ensure that all main figure and supplemental figure citations follow a uniform style.

Minor comments

Minor 1: Figure legends should be revised for clarity and consistency. In particular, there is redundancy between all figure descriptions in the Results section and the dedicated Figure legends section, and panel labeling (e.g., Figure 1 panels are labeled a-e in the Results but f-j in the legends, despite referring to the same figure).

Response: We thank the reviewer for noticing this error. We have thoroughly revised the figure legends to improve clarity and consistency. we reduced redundancy between the Results text and the dedicated Figure legends section, corrected inconsistent panel labeling, and carefully checked all main and supplemental figure citations to ensure that each panel is consistently labeled and referenced throughout the manuscript.

Minor 2: Some inconsistencies are present in the reporting of statistical analyses. In particular, thresholds for differential accessibility (e.g., log2 fold-change cutoffs of 1.5 vs. 0.1) differ between the Methods and figure legends, and statistical tests and multiple testing corrections are not always described consistently across sections. Thie should be clarified and standardized to improve reproducibility and clarity.

Response: We thank the reviewer for pointing out these inconsistencies. The different thresholds reflect two distinct analyses. Specifically, we now clearly distinguish peak-level differentially accessible peak (DAP) analysis from gene-level differential accessibility analysis. Peak-level DAPs were defined using $FDR < 0.05$ and $|log2FC| \geq 1.5$, as described in the Methods (revised manuscript, Methods, lines 514–519). In contrast, Figure 3D shows differential gene accessibility based on ArchR gene activity scores. For this gene-level volcano plot, genes with $FDR < 0.05$ and $|log2FC| > 0.1$ were highlighted for

visualization, and this threshold has now been explicitly stated in the Figure 3D legend (revised manuscript, Figure 3 legend, lines 280–285). This clarification explains why different log2 fold-change thresholds were used for peak-level and gene-level analyses. We have also reviewed and standardized the reporting of statistical tests and correction methods throughout the manuscript.

Minor 3: The Methods section contains several minor grammatical and formatting issues, including subject-verb agreement errors and missing spaces. Examples include:

1) Line 418: "Standard induction therapy were administered to all patients". This should be revised to "Standard induction therapy was administered to all patients", as "induction therapy" is singular. Additionally, the sentence should begin after a full stop rather than following a comma.

2) Line 426: "Quality control(QC) metrics were applied", missing space after "control".

3) Line 427: "criteria:(i) fewer than 1,000", missing space after colon.

4) Line 475: "false discovery rate FDR {less than or equal to} 0.05", missing parentheses around FDR.

5) Line 476: "motifs enrichment", it should be "motif enrichment".

Response: We thank the reviewer for carefully identifying these grammatical and formatting issues in the Methods section. We have corrected all indicated errors in the revised manuscript. In addition, we reviewed the Methods section for similar minor grammatical and formatting issues.

August 10, 2026

RE: Life Science Alliance Manuscript #LSA-2026-03746-TR

Dr. Boyu Cui
Anhui Institute of Medicine
Department of Medical Technology
No. 632 Furong Road, Hefei, Anhui, China
Hefei 230601
China

Dear Dr. Cui,

Thank you for submitting your revised manuscript entitled "Single-cell chromatin profiling reveals early relapse-associated epigenetic priming in pAML". As you will see, the reviewers are satisfied with no major requests. We invite you to comment on the significance of the relapse signatures measured for FLT3-ITD in the manner of your choosing.

It is not completely clear from the results and materials and methods sections whether the clinical samples analyzed for this study were already collected and sequenced for chromatin accessibility in your prior study Cui et al (2025). It appears the healthy controls are identical, although your study lists fewer pAML samples than the prior study. The Abstract, Results, and Materials and Methods sections must be explicit about this and refer to the prior study where necessary. We would be happy to publish your paper in Life Science Alliance pending these changes and final revisions necessary to meet our formatting guidelines.

MANUSCRIPT ORGANIZATION AND FORMATTING:

To avoid unnecessary delays in the acceptance and publication of your paper, please read the following information carefully. Full guidelines are available on our Instructions for Authors page, https://www.life-science-alliance.org/authors

Submission of a paper that does not conform to Life Science Alliance guidelines will delay the acceptance of your manuscript.

-Please upload all figure files individually including the supplementary figure files; all figure legends should only appear in the main manuscript file. Figures should be removed from main manuscript file.
-Please add an ORCID ID for secondary corresponding author--you should have received instructions on how to do so.
-Please add the X and Bluesky handles of your host institute/organization, as well as your own, and/or one of the authors, in our system.
-The titles in both the system and the manuscript file must be consistent with each other. Please change the title in our system to match the manuscript file.
-Please add your main, supplementary figure, and table legends to the main manuscript text after the references section.
-Please label Methods section as 'Materials and Methods'.
-If clinical samples were collected as part of this study, please include an ethics statement concerning the collection and use of human tissues.
-Please include accession numbers for the public datasets reanalyzed in this study from TCGA and the TARGET cohort in the Data Availability section.
-Please consult our manuscript preparation guidelines https://www.life-science-alliance.org/manuscript-prep and make sure your manuscript sections are in the correct order.
-Please add a callout for Figure S1A-C to your main manuscript text.

We encourage our authors to provide original source data, particularly uncropped/-processed electrophoretic blots and spreadsheets for the main figures of the manuscript. If you would like to add source data, we would welcome one PDF/Excel-file per figure for this information. These files will be linked as supplementary "Source Data" files.

It is Life Science Alliance policy that if requested, original data images must be made available to the editors. Failure to provide original images upon request will result in unavoidable delays in publication. Please ensure that you have access to all original data images prior to final submission.

If you are planning a press release on your work, please inform us immediately to allow informing our production team and scheduling a release date.

We welcome submissions of potential cover images for the issue of LSA in which your work would appear. If you have high

quality images associated with this work, please feel free to email these, with a caption, to the journal office.

LSA encourages authors to provide a 30-60 second video where the study is briefly explained. These videos will be appear embedded with the manuscript online at Life Science Alliance, and on social media to promote the published paper and authors (for examples, see https://docs.google.com/document/d/1-UWCfbE4pGcDdcgzcmiuJl2XMBJnxKYeqRvLLrLSo8s/edit?usp=sharing). Corresponding or first-authors are welcome to submit the video. Please submit only one video per manuscript. The video can be emailed to contact@life-science-alliance.org

FINAL FILES:

To upload the final version of your manuscript, please log in to your account: https://lsa.msubmit.net/cgi-bin/main.plex
You will be guided to complete the submission of your revised manuscript and to fill in all necessary information. Please get in touch in case you do not know or remember your login name.

The following items are required for acceptance.

-- An editable version of the final text (.DOC or .DOCX) is needed for copyediting (no PDFs).

-- High-resolution figure, supplementary figure and video files uploaded as individual files: See our detailed guidelines for preparing your production-ready images, https://www.life-science-alliance.org/authors

-- Summary blurb (enter in submission system): A short text summarizing in a single sentence the study (max. 200 characters including spaces). This text is used in conjunction with the titles of papers, hence should be informative and complementary to the title. It should describe the context and significance of the findings for a general readership; it should be written in the present tense and refer to the work in the third person. Author names should not be mentioned.

The license to publish form must be signed before your manuscript can be sent to production. A link to the license to publish form will be available to the corresponding author only. Please take a moment to check your funder requirements.

Reviews, decision letters, and point-by-point responses associated with peer-review at Life Science Alliance will be published, alongside the manuscript. If you do want to opt out of having the reviewer reports and your point-by-point responses displayed, please let us know immediately.

Thank you for your attention to these final processing requirements. Please revise and format the manuscript and upload materials as soon as you are able.

Thank you for this interesting contribution to the literature. We look forward to publishing your paper in Life Science Alliance.

Sincerely,

Tim Fessenden
Scientific Editor
Life Science Alliance

Reviewer #1 (Comments to the Authors (Required)):

This manuscript seeks to mine the authors' prior dataset to look at epigenetic priming in pediatric acute myeloid leukemia. Finding epigenomic programs at diagnosis that can predict outcomes is an important endeavor. The authors were responsive to my prior comments, and I believe this paper will be a resource for the field. I support publication.

Reviewer #2 (Comments to the Authors (Required)):

The provided revisions, and revised language, now support the major claims of the work. I support publication of this work. However, the authors might comment on why the signatures correlating with relapsed leukemia seem to be more impactful in the FLT3-ITD setting.

August 11, 2026

RE: Life Science Alliance Manuscript #LSA-2026-03746-TRR

Dr. Boyu Cui
Anhui Institute of Medicine
Department of Medical Technology
No. 632 Furong Road, Hefei, Anhui, China
Hefei 230601
China

Dear Dr. Cui,

Thank you for submitting your Research Article entitled "Single-cell chromatin profiling reveals relapse-related priming in pediatric acute myeloid leukemia". It is a pleasure to let you know that your manuscript is now accepted for publication in Life Science Alliance. Many thanks for your prompt and thorough responses to our the the Reviewers' final requests. Congratulations on this work which we agree will interest your colleagues concerned with pediatric AML and mechanisms of relapse.

The final published version of your manuscript will be deposited by us to PubMed Central upon publication.

Your manuscript will now progress through copyediting and proofing. It is journal policy that authors provide original data upon request.

Your article will publish open access upon publication under a CC-BY license.

Reviews, decision letters, and point-by-point responses associated with peer-review at Life Science Alliance will be published, alongside the manuscript. If you do want to opt out of having the reviewer reports and your point-by-point responses displayed, please let us know immediately.

IMPORTANT: If you will be unreachable at any time, please provide us with the email address of an alternate author. Failure to respond to routine queries may lead to unavoidable delays in publication.

Scheduling details will be available from our production department. You will receive proofs shortly before the publication date. Only essential corrections can be made at the proof stage so if there are any minor final changes you wish to make to the manuscript, please let the journal office know now.

DISTRIBUTION OF MATERIALS:
Authors are required to distribute freely any materials used in experiments published in Life Science Alliance. Authors are encouraged to deposit materials used in their studies to the appropriate repositories for distribution to researchers.

You can contact the journal office with any questions, contact@life-science-alliance.org

Again, congratulations on a very nice paper. I hope you found the review process to be constructive and are pleased with how the manuscript was handled editorially. We look forward to future exciting submissions from your lab.

Sincerely,

Tim Fessenden
Scientific Editor
Life Science Alliance
